# The CDK Subunit CKS2 Counteracts CKS1 to Control Cyclin A/CDK2 Activity in Maintaining Replicative Fidelity and Neurodevelopment

**DOI:** 10.1016/j.devcel.2012.06.018

**Published:** 2012-08-14

**Authors:** Mattia Frontini, Alexander Kukalev, Elisabetta Leo, Yiu-Ming Ng, Marcella Cervantes, Chi-Wai Cheng, Roman Holic, Dirk Dormann, Eric Tse, Yves Pommier, Veronica Yu

**Affiliations:** 1 Eukaryotic Chromatin Dynamics Group, MRC Clinical Sciences Centre, Imperial College London, Hammersmith Campus, Du Cane Road, London W12 0NN, UK; 2 Microscopy Facility, MRC Clinical Sciences Centre, Imperial College London, Hammersmith Campus, Du Cane Road, London W12 0NN, UK; 3 Department of Medical & Molecular Genetics, King's College London School of Medicine, Guy's Hospital, Level 8, Tower Wing, Great Maze Pond, London SE1 9RT, UK; 4 Laboratory of Molecular Pharmacology, Center for Cancer Research, NCI, 37 Convent Drive, Building 37, NIH, Bethesda, MD 20892-4255, USA; 5 Division of Haematology, Department of Medicine, The University of Hong Kong, Hong Kong; 6 Institute of Animal Biochemistry and Genetics, Slovak Academy of Sciences Moyzesova 61, 90028 Ivanka pri Dunaji, Slovakia

## Abstract

CKS proteins are evolutionarily conserved cyclin-dependent kinase (CDK) subunits whose functions are incompletely understood. Mammals have two CKS proteins. CKS1 acts as a cofactor to the ubiquitin ligase complex SCF^SKP2^ to promote degradation of CDK inhibitors, such as p27. Little is known about the role of the closely related CKS2. Using a *Cks2*^−*/*−^ knockout mouse model, we show that CKS2 counteracts CKS1 and stabilizes p27. Unopposed CKS1 activity in *Cks2*^*−/*−^ cells leads to loss of p27. The resulting unrestricted cyclin A/CDK2 activity is accompanied by shortening of the cell cycle, increased replication fork velocity, and DNA damage. In vivo, *Cks2*^−*/*−^ cortical progenitor cells are limited in their capacity to differentiate into mature neurons, a phenotype akin to animals lacking p27. We propose that the balance between CKS2 and CKS1 modulates p27 degradation, and with it cyclin A/CDK2 activity, to safeguard replicative fidelity and control neuronal differentiation.

## Graphical abstract

**Graphical Abstract:**

**Figure:**
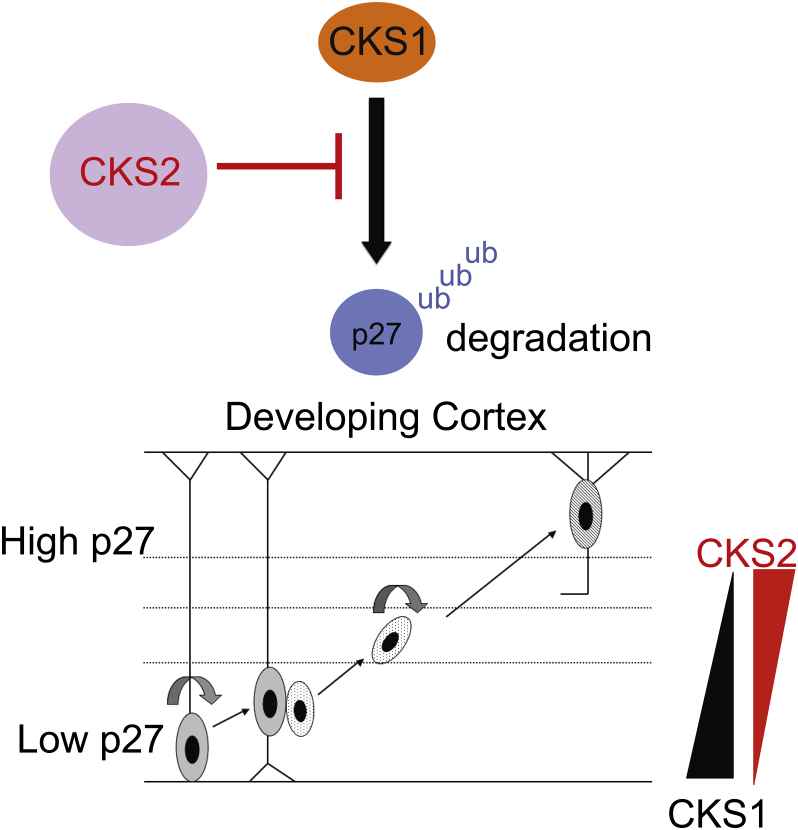

**Highlights:**

► Cks2 competes against Cks1 to limit p27 degradation ► Knockdown of Cks2 in MEFs leads to low p27 and hyperactivation of Cyclin A/CDK2 ► *Cks2*^−*/*−^ MEFs exhibit abnormal replication and G2/M checkpoint defects ► Differential expression of Cks1 and Cks2 in the fetal brain controls neurogenesis

## Teaser

The cyclin-dependent kinase subunit Cks1 is a SCF^Skp2^ cofactor that enhances p27 degradation. Frontini et al. find that its close paralog, Cks2, competes with Cks1 to protect p27 from degradation. In Cks2 knockout mice, low p27 levels cause defects in DNA replication and neural development.

## Introduction

The eukaryotic cell cycle comprises a series of precisely controlled events that coordinate cell growth and differentiation. Cell cycle progression is driven by oscillating activity of cyclin-dependent kinases (CDKs). CDK activity is determined by periodic expression of activating cyclins and counteracted by binding to CDK inhibitory proteins (CKIs), as well as a combination of activating and inhibitory CDK phosphorylation. Feedback regulation via ubiquitin-dependent proteasomal degradation of cyclins and CKIs controls CDK activity and transitioning between different cell cycle stages.

The CDK catalytic subunit CDK2 associates with cyclins E and A and is active from late G1 until the onset of mitosis (Pagano et al., 1993). Gene disruption in murine models indicates that CDK2 is not essential for survival (Berthet et al., 2003; Ortega et al., 2003), as CDK1 can compensate for its functions (Santamaría et al., 2007). However, acute inhibition of CDK2 in proliferating cells is not tolerated and has revealed a nonredundant and rate-limiting role for cyclin A-bound CDK2 (cyclin A/CDK2) in regulating the G1/S transition (Merrick et al., 2011). Cyclin A/CDK2 promotes firing of DNA replication origins and its deregulation leads to aberrant replication, accumulation of DNA damage and loss of DNA damage checkpoint control (Merrick et al., 2008; Tane and Chibazakura, 2009; Wheeler et al., 2008). CDK2 is also an important regulator of stem cell maintenance and differentiation. As an example, in the central nervous system, the length of G1 and S phase are instrumental in controlling cell cycle exit and neuronal maturation. Consistently, loss of CDK2 activity results in the depletion of neural stem cells (Caillava et al., 2011; Jablonska et al., 2007).

CKS (cyclin dependent-kinase subunit) proteins are evolutionarily conserved small proteins, which tightly associate with CDKs. Mammalian cells contain two CKS paralogs, CKS1 and CKS2, that share over 80% sequence identity. These bind to CDK2 and CDK1-containing CDK complexes during cell cycle phases when these are active (Egan and Solomon, 1998). CKS proteins are essential for cell proliferation as *Cks1* and *Cks2* double knockout mice are not viable (Martinsson-Ahlzén et al., 2008). The essential function of CKS proteins is still under debate. It could relate to its function in specifying multisite phosphorylation of a crucial subset of CDK substrates (Patra et al., 1999; Reynard et al., 2000; Kõivomägi et al., 2011). Alternatively, functions in transcription have been reported in yeast (Morris et al., 2003; Holic et al., 2010; Yu et al., 2005) and mammalian cells (Martinsson-Ahlzén et al., 2008; Westbrook et al., 2007). Recently, CKS proteins have been implicated in facilitating checkpoint-resistant cyclin A degradation by the APC in early mitosis (van Zon et al., 2010; Wolthuis et al., 2008). To date, it is poorly understood why higher eukaryotes possess two CKS proteins as most known CKS protein functions are shared between the two paralogs. Using the *Cks1* knockout mouse model, it was revealed that CKS1 facilitates S phase entry by accelerating degradation of the CDK2 inhibitor, p27 (Spruck et al., 2001). Accordingly, *Cks1*^−*/*−^ mice accumulate excess p27 and are small compared to wild-type littermates. Mechanistically, CKS1 acts as a cofactor for the E3 ubiquitin ligase complex SCF^SKP2^ in p27 ubiquitylation. CKS1 bridges the LRR domain in SKP2 and phosphorylated p27 and induces a conformational change in SKP2 for optimal substrate positioning (Hao et al., 2005; Sitry et al., 2002; Yao et al., 2006). Although CKS2 shares a similar CDK interaction site with CKS1, as well as the anion-binding pocket that recognizes phosphorylated p27, CKS2 lacks the residues in the N terminus of CKS1 that are required for interacting with SKP2 (Hao et al., 2005). Mice lacking *Cks2* do not show any overt phenotypes except sterility in both sexes (Spruck et al., 2003). This has been attributed to the absence of CKS1 expression in germ cells and thus cannot substitute for CKS2 function in these cells.

In this study, we have re-examined the role of CKS2. Although cells lacking CKS1 have elevated p27 and reduced cyclin A/CDK2 activity, we observe that the opposite is true in cells lacking CKS2. p27 stability is reduced, causing replication stress and DNA damage checkpoint failure due to increased cyclin A/CDK2 activity. In vivo, CKS2 is required to promote neuronal differentiation during brain development. We propose a model in which the balance of CKS2 and CKS1 regulates p27 stability to fine-tune CDK activity in maintaining replicative fidelity and controlling neuronal differentiation.

## Results

### CKS2 Differs from CKS1 in Controlling Cell-Cycle Progression

**Figure 1:**
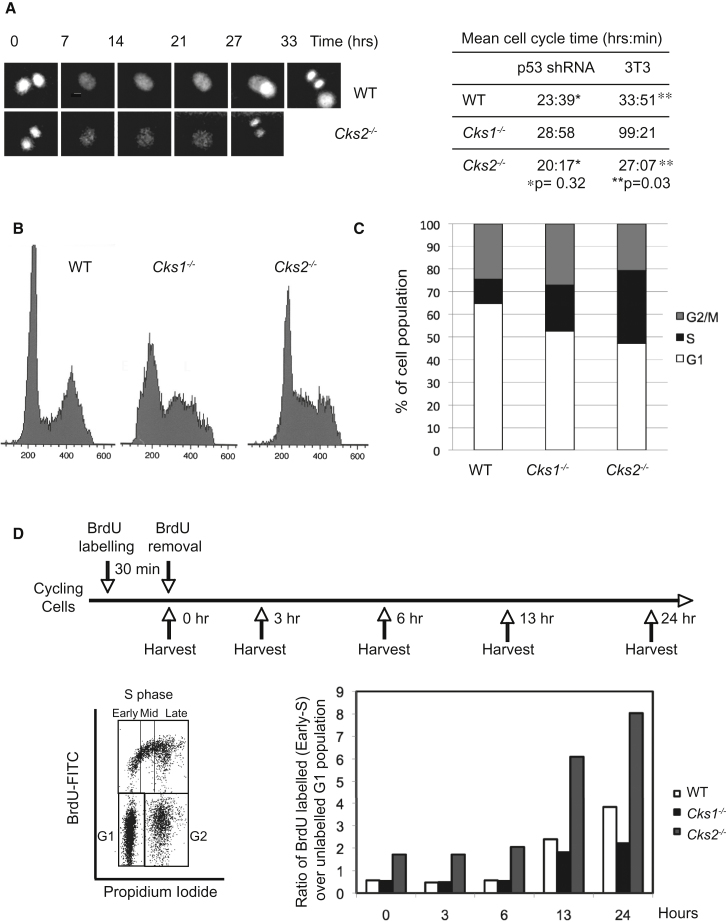
Accelerated Cell-Cycle Progression in *Cks2*^−/−^ MEFs (A) Time-lapse microscopy of asynchronously growing wild-type, *Cks2*^−/−^ and *Cks1*^−/−^ MEFs. MEFs were immortalized using shRNA against p53. Wild-type MEFs were infected with a retrovirus expressing histone H2B-GFP, *Cks2*^−/−^ and *Cks1*^−/−^ MEFs expressed histone H2B-mCherry. Wild-type and knockout MEFs were mixed and imaged. The time between two successive anaphases was recorded for at least 50 cells of each genotype; the mean is shown. An example of a wild-type and *Cks2*^−/−^ cell is shown. Alternatively, MEFs were immortalized using the 3T3 protocol, and cell cycle duration was measured as the duration from one cytokinesis to the next for at least 50 cells per genotype. (B) FACS analysis of DNA content to generate cell cycle profiles of asynchronously proliferating wild-type, *Cks2*^−/−^ and *Cks1*^−/−^ MEFs. (C) Quantification of the cell cycle populations by FACS. (D) Experimental design and example of the FACS pattern of wild-type MEFs at time 0, depicting the G1, BrdU-positive and G2 gates. The ratio of BrdU-positive cells with a G1 DNA content (labeled “early S”) over unlabeled G1 cells was followed over a 24 hr chase.

To investigate cell cycle progression in the absence of CKS2, we derived mouse embryonic fibroblasts (MEFs) from animals harboring a genomic *Cks2*^−/−^ deletion. We first assessed whether the absence of CKS2 had any effect on the overall duration of the cell cycle in spontaneously immortalized MEFs using the 3T3 protocol (Todaro and Green, 1963). Using live microscopy on MEFs grown in culture, we measured the time taken for these cells to divide (Figure 1A). We compared cells lacking CKS2 to wild-type controls and to cells lacking the closely related CKS1. As expected, due to an increased level of the CDK inhibitor p27 (Spruck et al., 2001), *Cks1*^−*/*−^ cells showed a long delay in cell cycle progression. Their overall cell cycle duration was almost three times that of wild-type cells. Surprisingly, *Cks2*^−*/*−^ cells divided significantly faster, with average cell cycle duration 15% shorter than wild-type cells. MEFs may display varying cell cycle lengths depending on their mode of immortalization. We therefore compared spontaneously immortalized MEFs to those independently immortalized at early passages by retrovirally delivered shRNA against p53 (Dickins et al., 2005), which often have shorter cell cycles. Although the measured cell cycle durations were thus consistently shorter, *Cks1*^−*/*−^ sh-p53 silenced MEFs still proliferated significantly slower and *Cks2*^−*/*−^ cells divided 15% faster than wild-type controls. This revealed that despite their similarity, CKS1 and CKS2 affect cell cycle progression in opposing manners.

To examine whether the duration of specific cell cycle stages was affected by the absence of CKS1 and CKS2, we examined cell cycle profiles of exponentially dividing cells by flow cytometric analysis of DNA content after staining with propidium iodide (Figure 1B). *Cks1*^−*/*−^ cells harbored largely widened G1 and S phase populations, consistent with a cell cycle delay at the G1/S transition due to elevated p27. *Cks2*^−*/*−^ cells showed a reduced G1 population and an increased fraction of cells in S phase, consistent with accelerated progression through the G1/S boundary. To substantiate this cell cycle analysis, we pulse-labeled cells with the thymidine analog bromodeoxyuridine (BrdU) and followed the fate of the labeled cells over the course of 24 hr (Figure 1D). In agreement with the flow cytometry profile, *Cks1*^−*/*−^ and *Cks2*^−*/*−^ cells both showed a higher than wild-type proportion of cells with positive BrdU uptake. *Cks1*^−/−^ cells showed twice and *Cks2*^−/−^ cells three times as many BrdU-incorporating cells compared to wild-type. Upon washout of the BrdU label, we analyzed how the ratio of labeled versus unlabeled G1 cells developed over time (Figure 1D). As the labeled cells progress from S phase through G2 and mitosis into the next G1 phase, the proportion of labeled versus unlabeled G1 cells increases. This increase occurred at a much faster rate in *Cks2*^−/−^ cells, compared to wild-type, suggestive not only of faster entry into S phase but also faster progression through G2 and mitosis. *Cks1*^−/−^ cells on the other hand, despite the initially high fraction of BrdU-incorporating cells, displayed only a very slow increase in the fraction of labeled over unlabeled G1 cells. This is consistent with delayed entry and slow progression through S phase of *Cks1*^−*/*−^ cells. Taken together, these results suggest that the increased S phase population in *Cks1*^−/−^ and *Cks2*^−/−^ cells arises for different reasons. *Cks1*^−/−^ cells progress slowly through S phase. In contrast, *Cks2*^−/−^ cells show a high proportion of S phase cells because of a fast G1/S transition and an overall shorter cell cycle.

### CKS2 Guards Replicative Fidelity

**Figure 2:**
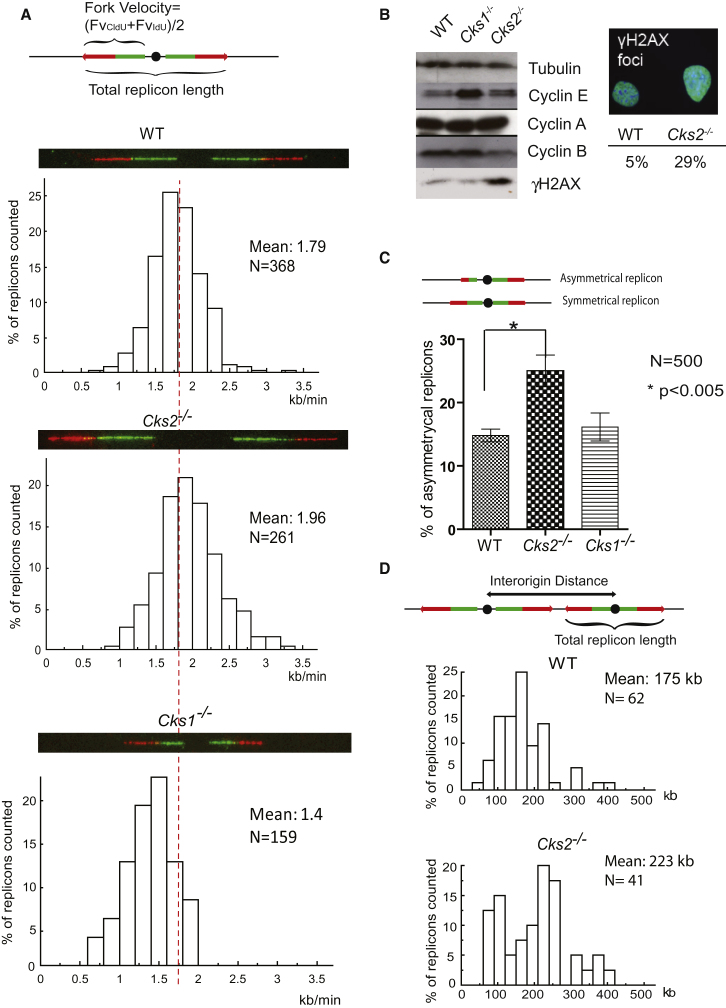
*Cks2*^−*/*−^ MEFs Harbor Replicative Abnormalities and DNA Damage (A) Molecular combing of DNA fibers from MEFs, sequentially labeled with IdU and CldU for 20 min each. Replication fork velocity was calculated by dividing the total length of two sequential IdU and CldU signals by pulse time. A typical DNA fiber track (IdU, green; CldU, red) is depicted together with a histogram of the fork velocity distribution (red line indicates mean fork velocity of wild-type). The distributions in both *Cks2*^−/−^ and *Cks1*^−/−^ MEFs were different from wild-type (p < 0.001, Mann-Whitney). (B) Left: Western blotting of extracts from wild-type (WT), *Cks1*^−/−^ and *Cks2*^−/−^ MEFs using the indicated antibodies. Right: Immunofluoresence demonstrating γH2AX foci in nuclei of asynchronously dividing MEFs. Representative image upon which threshold was set was shown. To determine the percentage of nuclei positive for γH2AX staining, 1,000 nuclei were counted per genotype. (C) Quantification of fork asymmetry between left and right forks. Mean and standard deviation of three independent experiments is shown in which a total of 500 forks were analyzed for each genotype. (D) Histogram of interorigin distances in wild-type and *Cks2*^−/−^ MEFs.

To obtain further insight into S phase progression in *Cks2*^−/−^ cells, we examined replication dynamics using molecular DNA combing. This technique measures the speed of individual replication forks as well as the frequency of replication initiation events. Asynchronously growing cells were sequentially pulse-labeled with two thymidine analogs, iododeoxyuridine (IdU) and chlorodeoxyuridine (CldU), for 20 min each. DNA was combed on silanized glass slides, and stretches of newly synthesized DNA were visualized by immunofluorescence staining of IdU and CldU (Figure 2A, shown as green and red tracks, respectively). Velocity of individual replication forks was calculated by dividing the length of each fluorescent segment (1 μm ≈2 kb) by the time of the pulse (20 min), as previously described (Conti et al., 2001; Seiler et al., 2007). This analysis revealed that *Cks2*^−*/*−^ cells showed a small but significant and reproducible (p < 0.001, Mann-Whitney) increase in fork velocity compared to wild-type cells. The mean fork velocity in *Cks2*^−*/*−^ cells was 1.96 kb/min versus 1.79 kb/min in wild-type cells. The opposite was seen in *Cks1*^−*/*−^ cells, where the fork velocity was greatly reduced to a mean of 1.4 kb/min. The slow fork progression could in part explain the slower progression through S phase of *Cks1*^−*/*−^ cells.

Changes in fork velocity may impact on replicative fidelity (Anglana et al., 2003; Courbet et al., 2008). We therefore analyzed whether the observed changes in replication fork speed in *Cks2*^−*/*−^ and *Cks1*^−*/*−^ cells were associated with DNA damage (Figure 2B). Western blotting indicated the presence of DNA damage in asynchronously growing *Cks2*^−*/*−^ cells, as seen by increased levels of phosphorylated histone H2AX (γH2AX). This was further confirmed by counting the number of nuclei harboring positive γH2AX foci in asynchronously growing MEFs (Figure 2B, right). *Cks2*^−*/*−^ MEFs had almost 6-fold more cells with γH2AX-positive nuclei. The presence of γH2AX is suggestive of double-strand DNA breaks, a common consequence of stalled or collapsed replication forks during S phase. The increased fork velocity could be a reason for replication fork damage. In *Cks1*^−*/*−^ cells, where fork velocity was decreased and the overall cell cycle duration prolonged, no indication of DNA damage was observed. Our western blot analysis also provided further insight into the altered cell cycle profiles of *Cks2*^−*/*−^ cells. Asynchronously growing *Cks2*^−*/*−^ cells had lower cyclin B levels, consistent with fast progression through the G2 and mitosis (compare Figure 1C). *Cks1*^−*/*−^ cells displayed normal cyclin B but elevated cyclin E levels, as previously reported (Spruck et al., 2001). This has been postulated to compensate for increased p27 in these cells. Cyclin A levels were comparable across all three genotypes.

One possible explanation for increased γH2AX levels in *Cks2*^−*/*−^ cells could be stalled or broken DNA replication forks. To address whether there was evidence of replication fork stalling, we analyzed 500 replicons in wild-type, *Cks1*^−*/*−^ and *Cks2*^−*/*−^ DNA fibers by molecular combing and scored the fraction of bidirectional forks with asymmetries in fork progression during the second pulse (Figure 2C). Asymmetries between the left and right arms arise if one fork continues to replicate, whereas the other fork slows down or stalls. *Cks2*^−*/*−^ cells displayed almost twice the number of such asymmetries when compared to wild-type cells. *Cks1*^−*/*−^ cells did not demonstrate statistically significant changes in the number of asymmetrical replicons compared to wild-type, consistent with the lack of γH2AX induction in these cells. These findings are consistent with the possibility that DNA damage in *Cks2*^−*/*−^ cells arises because of stalled or broken replication forks.

In response to stalled replication forks, it is thought that dormant replication origins in the vicinity are activated to aid fork recovery in order to prevent persistence of replication intermediates or unreplicated regions into mitosis (Ge and Blow, 2010; Kawabata et al., 2011). Examination of active origin distribution in *Cks2*^−*/*−^ cells revealed that the mean interorigin distance was increased to 223 kb, compared to 175 kb in wild-type cells (Figure 2D). This increase is expected from the observed elevated fork velocity, as fork velocity and interorigin distances show a linear correlation (Conti et al., 2007; Seiler et al., 2007). Despite the mean increase in interorigin distance, we observed a population of shorter interorigin distances, below 100 kb, in *Cks2*^−*/*−^ cells, which was not present in the wild-type control. The appearance of this subset of short interorigin distances is in keeping with the firing of dormant origins in the vicinity of stalled replication forks.

### CKS2 Is Required for Cell-Cycle Arrest in Response to DNA Damage

Replication fork stalling and associated double-strand DNA breaks typically trigger a DNA damage response (DDR) with temporary delay to cell cycle progression (reviewed in Bensimon et al., 2011). Considering our evidence for fork stalling and DNA breaks, it was surprising that cell cycle progression appeared accelerated rather than delayed in *Cks2*^−*/*−^ cells. This raised the question whether DDR was functional. To address this, we investigated the response of *Cks2*^−/−^ cells to exogenous DNA damage.

**Figure 3:**
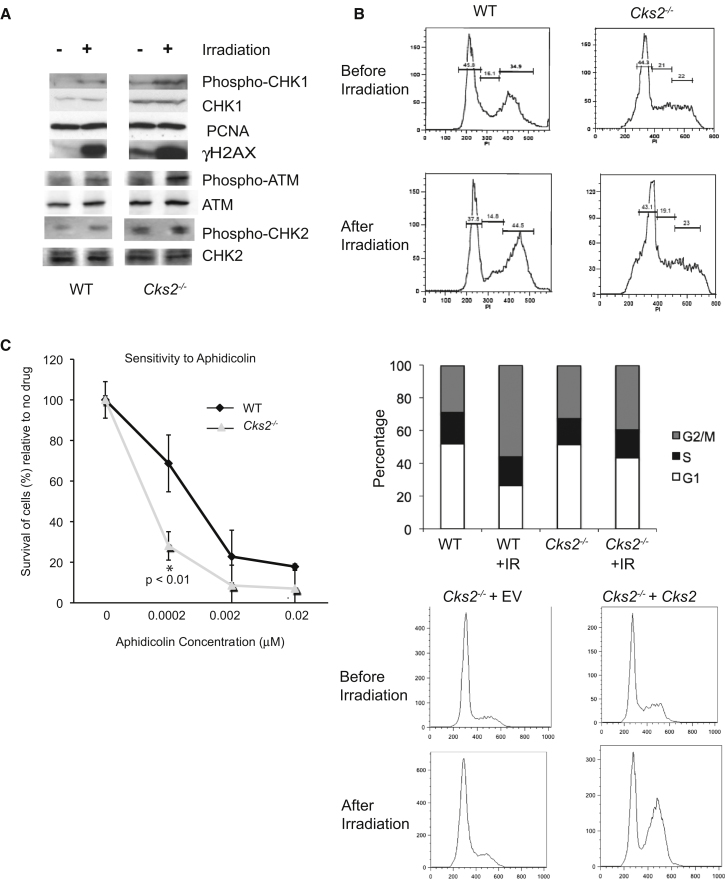
Defective DNA Damage Response in the Absence of *Cks2* (A) Proteins extracted from wild-type (WT) and *Cks2*^−/−^ MEFs before and after irradiation were subjected to western blotting using the indicated antibodies. (B) Failure of cell cycle arrest despite checkpoint kinase activation. Top: Wild-type and *Cks2*^−/−^ MEFs were exposed to 5 Gy of γ-irradiation. After 5 hr, the cell cycle distribution was analyzed by FACS analysis of DNA content. Bottom: Reconstitution of *Cks2*^−/−^ MEFs with wild-type *Cks2* or empty vector (EV). (C) Sensitivity of *Cks2*^−/−^ MEFs to replication stress. Wild-type and *Cks2*^−/−^ cells were plated at equivalent densities and exposed to increasing doses of aphidicolin. Each data point represents the mean and standard error of three experimental replicates (^∗^ indicates p < 0.01).

Asynchronously growing wild-type or *Cks2*^−/−^ MEFs were irradiated with a moderate dose (5 Gy) of γ-irradiation from a ^137^Cs source. Five hours later, a strong γH2AX signal could be detected by western blotting in wild-type cells (Figure 3A). Likewise, in *Cks2*^−/−^ cells, despite the endogenous presence of γH2AX in unirradiated cells, γH2AX was further augmented after irradiation. We conclude that early events of DNA damage detection that result in activation of ATM/ATR kinase and H2AX phosphorylation were largely functional in both wild-type and *Cks2*^−/−^ cells (Figure 3A).

The next molecular event in the DDR pathway we analyzed was activation of the downstream effector checkpoint kinase CHK1. In contrast to wild-type MEFs, *Cks2*^−/−^ cells showed a detectable level of spontaneous CHK1 activation in the absence of exogenous DNA damage (detected using a phosphospecific S317 CHK1 antibody) (Figure 3A) (Wilsker et al., 2008). This is consistent with the presence of endogenous DNA damage as a consequence of replication abnormalities. Upon irradiation, phospho-CHK1 accumulated both in wild-type and *Cks2*^−/−^ cells, suggesting that much of the DDR up to activation of effector checkpoint kinases is operational in the absence of CKS2. Similar findings were obtained when we assessed phosphorylation of CHK2 after irradiation (Figure 3A).

In response to CHK1 and CHK2 activation, wild-type cells accumulate in G2 phase of the cell cycle because of inhibition of G2/M transition. However, despite intact DDR signaling, there was no accumulation of *Cks2*^−/−^ cells in the G2 phase of the cell cycle in response to irradiation (Figure 3B). This checkpoint defect was reversed when *Cks2*^−*/*−^ cells were reconstituted with a vector expression wild-type *Cks2* (Figure 3B, lower panels). Therefore, cell cycle control network at the G2/M transition appears to be unresponsive to CHK1 and CHK2 signaling in *Cks2*^−*/*−^ cells. This could explain how *Cks2*^−/−^ cells accumulate DNA damage but nevertheless progress through an accelerated cell cycle.

Mutations in genes involved in DDR frequently lead to cell sensitivity to DNA damaging agents (Polo and Jackson, 2011). We tested the sensitivity of *Cks2*^−*/*−^ cells to an agent that induces replicative stress by inhibiting replicative DNA polymerases, aphidicolin. In line with our observations of defective cell cycle arrest in response to replication-induced DNA damage, *Cks2*^−*/*−^ cells displayed increased sensitivity to low doses of aphidicolin (Figure 3C). This confirms the requirement of CKS2 for an intact response to replication-induced DNA damage. A recent screen for DDR genes, carried out in a human cell line using γ-irradiation to induce damage (Cotta-Ramusino et al., 2011), found that silencing of *Cks2* using siRNA targeting resulted in a “medium” suppression of the DDR pathway, consistent with our results.

### Increased Cyclin A/CDK2 Activity in *Cks2*^−*/*−^ Cells

**Figure 4:**
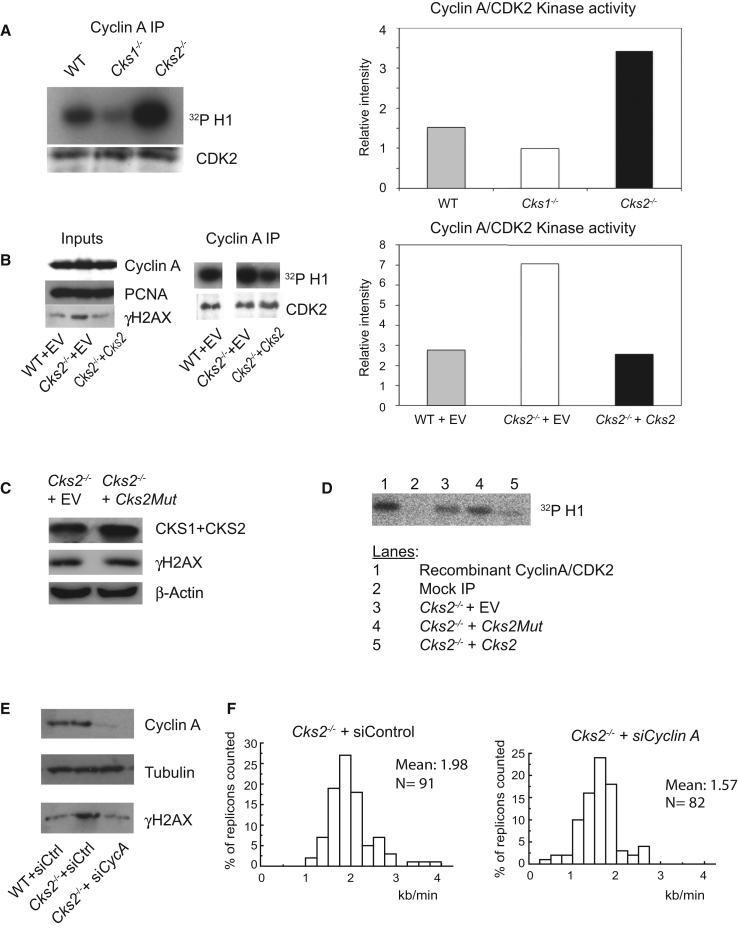
Increased Cyclin A/CDK2 Activity in *Cks2*^−/−^ MEFs (A) Cyclin A was immunoprecipitated from cell extracts obtained from wild-type, *Cks2*^−/−^ and *Cks1*^−/−^ MEFs. In vitro kinase activity using recombinant histone H1 as a substrate was measured. The kinase activity was normalized to the levels of CDK2 recovered in each sample. (B) Wild-type CKS2 reverses increased cyclin A/CDK2 activity and DNA damage in *Cks2*^−/−^ MEFs. Protein levels and cyclin A-associated kinase activity were measured in wild-type or *Cks2*^−/−^ MEFs, infected with either an empty retroviral vector (EV) or a vector containing *Cks2*. (C and D) A *Cks2* mutant that harbors mutations in the anion-binding pocket that abrogates its binding with phosphosubstrates (denoted *Cks2Mut*) cannot rescue the *Cks2*^−*/*−^ phenotype. (C) Western blot showing γH2AX levels in *Cks2*^−/−^ MEFs, infected with either an empty retroviral vector (EV) or a vector containing *Cks2Mut*. (D) CyclinA/CDK2 activity using recombinant histone H1 as a substrate. Cyclin A was immunoprecipitated in *Cks2*^−/−^ MEFs reconstituted with either an empty retroviral vector (EV), a vector containing *Cks2* (*Cks2*), or a vector containing the *Cks2-Mut*. Kinase activity was normalized to the levels of CDK2 recovered in samples used in lanes 3–5. (E) DNA damage in *Cks2*^−/−^ MEFs is due to increased cyclin A/CDK2 activity. *Cks2*^−/−^ MEFs were infected with retroviruses expressing control or cyclin A siRNAs. Whole-cell extracts were prepared and analyzed by western blotting as indicated. (F) Increased replication fork speed in *Cks2*^−/−^ MEFs due to increased cyclin A/CDK2 activity. Histogram of fork velocity in *Cks2*^−/−^ MEFs, infected with control siRNA or siRNA against cyclin A. Fork speed was decreased after cyclin A depletion, p < 0.001 (Mann-Whitney). See also Figure S1.

Cell cycle arrest in G2 in response to DNA damage requires inhibition of CDK2-associated kinase activity. The inability of *Cks2*^−*/*−^cells to impose a G2 arrest, despite intact CHK1 and CHK2 signaling pathways, suggests that CDK2 inhibition was compromised. To investigate this, we initially measured cyclin A-associated CDK2 activity in asynchronously proliferating MEFs. We immunoprecipitated cyclin A from wild-type, *Cks2*^−/−^ and *Cks1*^−/−^ cells and performed an in vitro kinase activity assay using histone H1 as a substrate (Figure 4A). Comparable amounts of CDK2 were precipitated in each case. As reported, probably because of increased p27 levels, cyclin A/CDK2 activity was reduced in *Cks1*^−*/*−^ cells (Spruck et al., 2001). In contrast, *Cks2*^−*/*−^ cells displayed markedly elevated cyclin A/CDK2 activity. Interestingly, cyclin E/CDK2 activity was not affected in *Cks2*^−*/*−^ cells (Figure S1B, available online).

To confirm that elevated cyclin A/CDK2 activity was a direct consequence of *Cks2* deletion, we reconstituted the *Cks2*^−*/*−^ cell line with a retroviral construct expressing *Cks2* (Figures 4B and 4D). This corrected cyclin A/CDK2 activity in *Cks2*^−/−^ cells downward to wild-type levels. Restoration of *Cks2* also prevented the accumulation of γH2AX in these cells. Apart from binding the CDKs, CKS proteins harbor a conserved anion-binding domain that binds phosphosubstrates. We next examined whether the effect of CKS2 on cyclin A/CDK2 activity is dependent on the ability of CKS2 to bind phosphosubstrates. Triple mutations in the anion-binding pocket of CKS proteins (K11E, S51E, and R71A) are known to abrogate the ability of CKS proteins to bind phosphoproteins based on structural studies (Bourne et al., 1996). The triple mutant (hereby denoted as *Cks2Mut*) was unable to correct γH2AX levels (Figure 4C) or cyclin A/CDK2 hyperactivity in *Cks2*^−*/*−^ cells (Figure 4D), suggesting that CKS2 binds a phosphorylated substrate to control cyclin A/CDK2 activity.

We next examined whether the replication and DNA damage phenotypes observed in *Cks2*^−/−^ cells could be explained by increased cyclin A/CDK activity. We employed siRNA against cyclin A to reduce its protein levels (Figure 4E). Reduced cyclin A levels in *Cks2*^−/−^ cells entirely reverted increased γH2AX back to wild-type levels. This suggests that DNA damage in *Cks2*^−/−^ cells was caused by unrestrained cyclin A/CDK2 activity. If it is correct that DNA damage in *Cks2*^−/−^ cells stemmed from accelerated DNA replication fork progression, we would expect the enhanced fork velocity to be corrected by cyclin A knockdown. We therefore measured replication fork velocity in *Cks2*^−/−^ cells, treated with control or cyclin A siRNA (Figure 4F). Control siRNA-treated cells showed accelerated forks, moving with a mean velocity of 1.98 kb/min, consistent with our previous measurements. Cyclin A downregulation in these cells caused a reduction of mean fork speed to 1.57 kb/min, somewhat below what we observed in wild-type cells. Similarly, when we applied the CDK inhibitor roscovitine to *Cks2*^−/−^ cells, we observed a reduction in γH2AX levels as well as replication fork velocity (Figure S1A).

Together, these observations suggest that replication fork progression is sensitive to the levels of cyclin A/CDK2 activity and that replication abnormalities and DNA damage observed in *Cks2*^−*/*−^ cells were consequences of increased cyclin A/CDK2 activity. Moreover, this is dependent on the intactness of the phosphobinding pocket of CKS2.

### CKS2 Is Required to Maintain p27 Levels

CKS1 acts as an activating cofactor of SKP2 to facilitate ubiquitylation and subsequent proteasome-mediated degradation of p27 by recruiting SKP2 through a unique N-terminal extension that is not shared with CKS2 (Hao et al., 2005). CKS2 is therefore unable to interact with SKP2 and cannot substitute for CKS1 in promoting p27 degradation (Hao et al., 2005; Sitry et al., 2002; Spruck et al., 2001). In contrast, the anion-binding pocket through which CKS proteins interact with phosphorylated p27 is conserved in CKS2. Given that the ability of CKS2 to control cyclin A/CDK2 activity is dependent on its phosphoprotein binding domain (Figures 4C and 4D), we envisioned the possibility that CKS2 competes with CKS1 for binding to phosphorylated p27 and thereby could act as a buffer that protects p27 from degradation.

**Figure 5:**
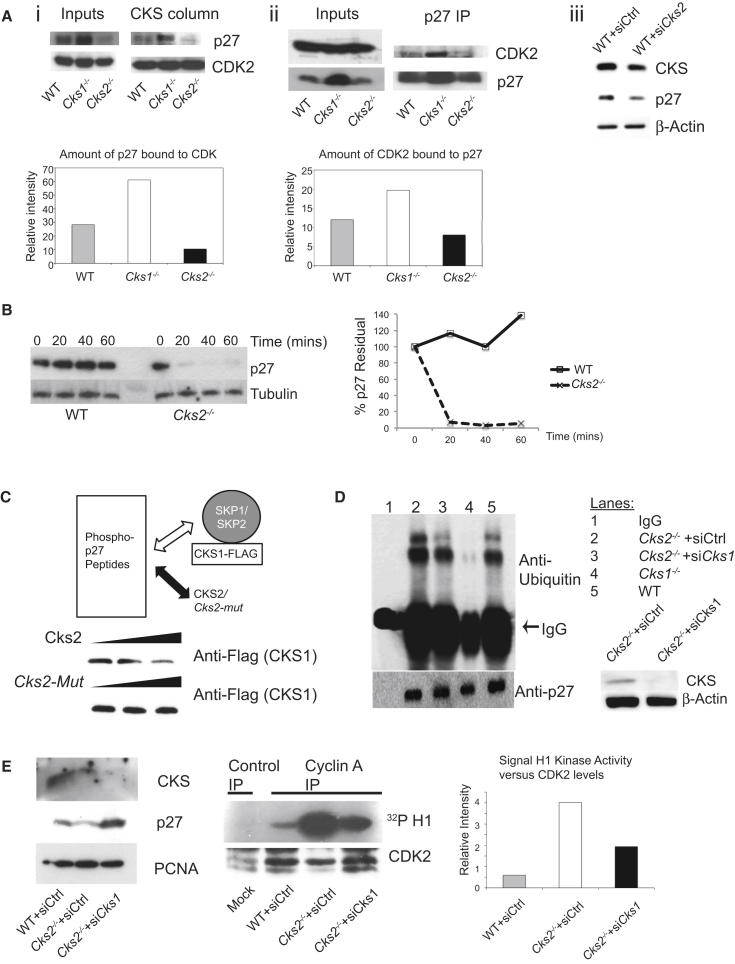
Increased p27 Degradation in *Cks2*^−/−^ MEFs Due to Unopposed CKS1 Action (A) (Ai) Cell extracts from wild-type, *Cks2*^−/−^ and *Cks1*^−/−^ MEFs were analyzed by western blotting. CDK complexes were pulled down using a CKS1 affinity column. Bound p27 was quantified and normalized to the levels of recovered CDK2. (Aii) As in (Ai), except that p27 was immunoprecipitated using an anti-p27 antibody. CDK2 associated with p27 was quantified and normalized to the p27 signal. (Aiii) Silencing *Cks2* in wild-type MEFs decreases p27 levels. Wild-type MEFs (WT) were infected with control siRNA or siRNA against *Cks2*. Western blotting demonstrating total CKS1 and CKS2 levels using an antibody that recognizes both CKS1 and CKS2 (denoted CKS), p27 levels, and actin. (B) Half-life of p27 in wild-type, *Cks2*^−/−^ and *Cks1*^−/−^ MEFs synchronized with nocodazole release was determined after addition of cyclohexamide and western blotting against p27. Tubulin served as a loading control. (C) In vitro phospho-p27 binding assay. Top: Schematic experimental design. Synthetic biotinylated phospho-p27 peptides were immobilized on a streptavidin column. Recombinant CKS1-Flag fusion protein was incubated with the affinity column either alone (left lanes) or premixed with wild-type recombinant CKS2 (top row) or mutant *CKS2* (that cannot bind phosphorylated p27 (*Cks2-Mut*)) in a 1:0.5 (CKS1:CKS2) or 1:1 (CKS1:CKS2) ratio in the presence of recombinant SKP1/SKP2 complexes. After stringency washes and elution, western blotting against the Flag epitope was carried out to determine whether CKS2 or *Cks2-Mut* were capable of competing with CKS1 for binding to phosphorylated p27 peptides. (D) CKS1-dependent cellular p27 ubiquitylation is increased in *Cks2*^−*/*−^ MEFs. p27 was immunoprecipitated from MEFs and western blotting was performed against ubiquitin to assess the level of p27 ubiquitylation in wild-type (WT), *Cks1*^−*/*−^ or *Cks2*^−*/*−^ MEFs. *Cks2*^−*/*−^ MEFs were infected with either a control siRNA (siCtrl) or siRNA against *Cks1*. (E) siRNA-mediated silencing of *Cks1* in *Cks2*^−/−^ MEFs restores wild-type p27 and cyclin A-associated kinase activity. The “CKS” antibody used for western blotting recognizes both CKS1 and CKS2. Cyclin A-associated kinase activity was measured and normalized against the amount of CDK2 precipitated. See also Figure S2.

To address whether this was the case, we compared p27 levels in wild-type, *Cks2*^−/−^ cells and *Cks1*^−/−^ cells (Figure 5A). As expected, we detected elevated p27 levels in *Cks1*^−/−^ cells. Consistent with the possibility that CKS2 protects p27 from degradation, p27 levels were lower in extracts derived from *Cks2*^−/−^ cells. Using a CKS affinity column, we pulled down CDK complexes from these extracts. This experiment showed that about twice more p27 was associated with CDK complexes in *Cks1*^−/−^ cells (Figure 5Ai). Compared to wild-type cells only a third of CDK was bound to p27 in *Cks2*^−/−^ cells. This finding was confirmed using an alternative method. We immunoprecipitated p27 from the cell extracts and specifically probed for CDK2 bound to it (Figure 5Aii). Again p27 was less abundant in *Cks2*^−*/−*^ extracts. Despite efficient pull down of p27 by immunoprecipitation, less p27 was bound to CDK2 in *Cks2*^−/−^ cells compared to wild-type or *Cks1*^−/−^ cells. To further confirm the direct relationship between p27 levels and CKS2, we employed siRNA to acutely knockdown *Cks2* in wild-type cells (Figure 5Aiii). p27 levels decreased after transient silencing of *Cks2* in wild-type cells, affirming our finding in *Cks2*^−*/*−^ MEFs. Lower levels of p27 bound to CDK2 in *Cks2*^−/−^ cells could explain the higher level of cyclin A/CDK2 activity that we observed. If our hypothesis was correct that CKS2 protects p27 from degradation, its rate of destruction should be accelerated in *Cks2*^−/−^ cells. We tested this by assaying p27 decay after cycloheximide treatment to inhibit protein synthesis (Figure 5B). To control for differences in cell cycle profiles, MEFs were synchronized by release from nocodazole. Compared to wild-type cells, p27 had a greatly reduced half-life in the *Cks2*^−/−^ background (over 50% of p27 was degraded in *Cks2*^−/−^ cells within 15 min, whereas in the wild-type, p27 remained largely stable).

To show that CKS2 competes with CKS1 for binding to phosphorylated p27, we carried out an in vitro binding assay modified from Hao et al. (2005) (Sitry et al., 2002). Recombinant CKS1 fused to the FLAG epitope binds the phosphorylated p27 peptides in the presence of the SKP1/SKP2 complex (Figure 5C). Recombinant CKS2 when added at a 1:1 ratio to CKS1 efficiently reduced binding of CKS1 to phosphorylated p27. The triple *CKS2* mutant (*Cks2-Mut*), which was incapable of binding phosphorylated p27, did not compete with CKS1 for binding. If our model was correct that CKS2 competes with CKS1 for p27 binding and protects it from degradation, we would expect *Cks2*^−/−^ cells to have increased ubiquitylation of p27. We examined cellular ubiquitylation of p27 by immunoprecipitation of p27 in MEFs in the presence of the proteasome inhibitor MG132 and looked for the level of ubiquitylation (Figure 5D). In *Cks1*^−/−^ cells, p27 ubiquitylation was markedly reduced, in agreement with published literature (Spruck et al., 2001). On the other hand, *Cks2*^−/−^ cells showed increased ubiquitylation, consistent with the observed decreased stability (Figure 5B). We predict the loss of p27 in *Cks2*^−/−^ cells to be due to unopposed action of CKS1. Applying siRNA to *Cks1*, we found reduced ubiquitylation of p27 in cells lacking *Cks2* (Figure 5D). To further demonstrate that reducing CKS1 levels in cells lacking CKS2 had functional significance, we tested whether restoration of p27 levels could reverse the increased cyclin A/CDK2 activity. By using siRNA against *Cks1* in *Cks2*^−/−^ cells (Figure 5E), we found that decreasing *Cks1* expression in the *Cks2*^−*/*−^ background restored p27 levels and reduced cyclin A/CDK2 activity. Similarly, transient overexpression of p27 in *Cks2*^−*/*−^ cells also reduced cyclin A/CDK2 activity (Figure S2). These observations support the possibility that decreased p27 in *Cks2*^−/−^ cells is due to unrestrained CKS1 activity and that CKS2 and CKS1 together work to balance cellular p27 levels.

### CKS Proteins Control Neuronal Differentiation

**Figure 6:**
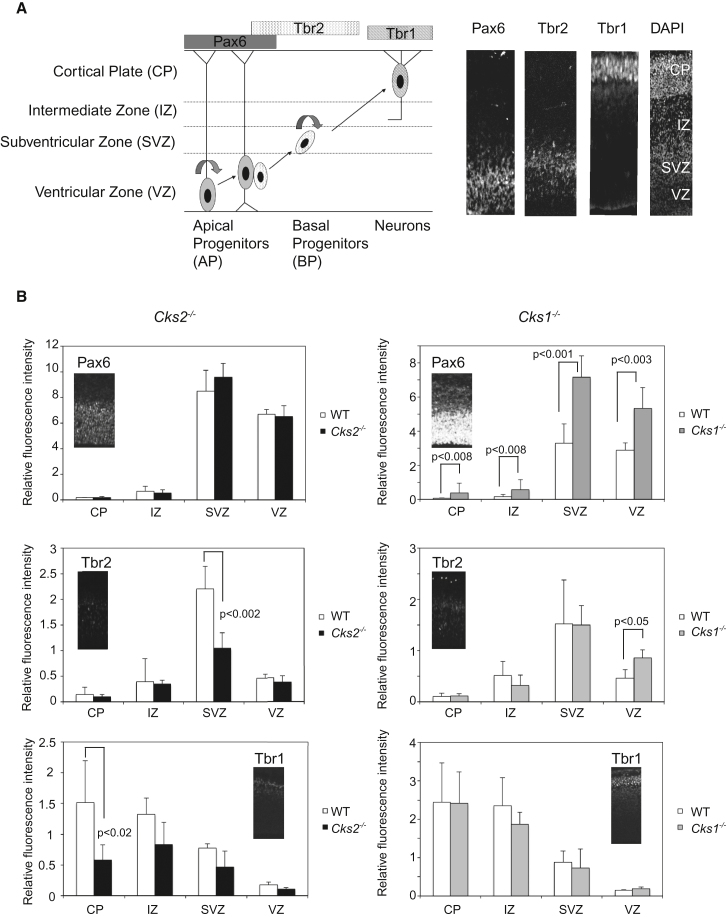
Neuronal Maturation Defects in *Cks2*^−/−^ and *Cks1*^−/−^ Mice (A) Schematic representation of neuronal maturation in the developing cortex. Neuronal stem cells reside in the ventricular zone (VZ) on the neuroepithelium that lines the ventricles. As neurons mature, they sequentially express the markers Pax6, Tbr2, and then Tbr1 as they migrate outward toward the cortical plate (CP). Right: Representative immunofluorescence staining of an E13.5 cortex in a wild-type mouse. (B) Quantification of Pax6, Tbr2, and Tbr1 signals in E13.5 *Cks2*^−/−^ and *Cks1*^−/−^ brains compared to wild-type. The respective intensity of signals from specific antibody staining was normalized against the signal of DAPI in each sample. Each data point is an average of three experimental replicates (three fetal brains of equivalent coronal sections). Error bars represent standard deviation. Representative images from each genotype are inserted next to the graphs. See also Figure S3.

Both SKP2 and p27 have been implicated in the regulation of cell differentiation and organogenesis during mammalian development. In particular, overexpression or depletion of either protein has been linked to abnormalities in neurogenesis (Nguyen et al., 2006b). Given our results in cultured cells, which implicate CKS2 in balancing p27, we wondered whether CKS2 might play a role in neurogenesis during mouse development. In the embryonic neocortex, progenitor cells divide in the ventricular (VZ) and subventricular zones (SVZ; Figure 6A). As neurons mature, they migrate outward from the VZ and SVZ to the intermediate zone (IZ) and finally form laminar layers in the cortical plate (CP). As progenitors mature, they sequentially express a series of transcription factors, which display a zonal pattern of expression from the VZ to the CP (Pax6 → Tbr2 → Tbr1, Figure 6A).

We examined fetal brains from day E13.5 embryos in the *Cks2*^−*/*−^ and *Cks1*^−*/*−^ backgrounds and compared them with wild-type littermates (Figure 6B). *Cks2*^−*/*−^ brains maintained a comparable Pax6-positive population of apical progenitors compared to wild-type littermates. However, there was a marked loss of Tbr2-positive cells, consistent with a reduction in the basal progenitor population. Consequently, there was also a reduction of Tbr1-positive mature neurons in upper cortical layers. This distribution is suggestive of a defect in neuronal differentiation, reminiscent of the defect seen in p27 knockout brains (Nguyen et al., 2006a, 2006b). Consistent with our findings in MEFs, we found that cell cycle duration was shortened in cortical progenitors in the *Cks2*^−*/*−^ brain (highlighted by an increased proportion of cells staining positive for the mitotic marker phosphohistone H3 after animals were pulsed-labeled with BrdU for 24 hr; Figure S3C).

We observed a different outcome in *Cks1*^−*/*−^ mice. There was an overall increase in the Pax6 population in all zones. The Tbr2-positive population was increased in the VZ, whereas the subsequent Tbr1-positive population remained unaltered. The increase in Tbr2-positive cells may represent premature neuronal differentiation in the VZ secondary to elevated p27. We examined p27 expression and found that this was indeed increased in the *Cks1*^−*/*−^ cortex, particularly in the VZ (Figure S3B). The increase in p27 levels was sustained up to the IZ but not at the CP. High p27 staining at the VZ was an unexpected finding, as p27 is not normally expressed at the VZ, where cells are actively dividing. We suspect, therefore, that the high level of Pax6-positive cells in the *Cks1*^−*/*−^ cortex may represent an increased population of nondividing progenitor cells.

To demonstrate that the defect in neurodevelopment was directly related to deletion of CKS proteins in the developing brain, we checked that both CKS2 and CKS1 were expressed in the developing cortex (Figure S3A). CKS1 expression was particularly enriched at the VZ in the E13.5 cortex (CKS2 expression at this layer is absent). CKS2, however, was expressed in the SVZ and increased in the IZ, where CKS1 expression decreases. In the CP, both proteins were expressed (though CKS2 levels are lower than CKS1).

Taken together, these results lead us to propose a model where in the normal developing brain, high CKS1 expression in the VZ encourages rapid degradation of p27, thus encouraging progenitor cell proliferation. As neurogenesis progresses through the cortical layers into the IZ, CKS2 expression increases and CKS1 expression decreases, promoting p27 accumulation and neuronal differentiation and migration toward the cortical surface. In the CP, where mature neurons reside, both CKS proteins are expressed. We also observed this pattern of expression in certain areas of the adult brain (unpublished data). The functional significance of this is yet unknown and further studies are currently warranted.

## Discussion

### CKS2 Antagonizes CKS1 in Controlling p27 Levels

**Figure 7:**
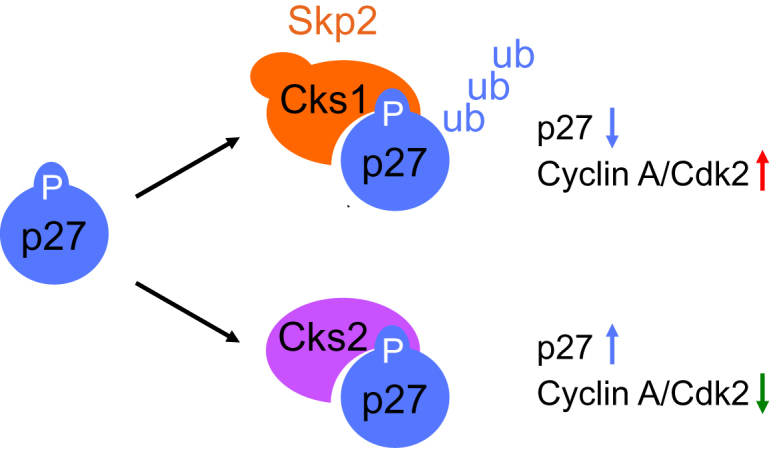
Model Depicting How CKS2 Antagonizes CKS1 in the Control of p27 CKS1 mediates recognition of phopshorylated p27 by the SCF^SKP2^ and promotes its ubiquitylation and destruction. CKS2 shares the anion pocket that recognizes p27 with CKS1 but not the N-terminal residues that recruit SKP2. CKS2 competes with CKS1 for binding to p27 and protects p27 from degradation.

Through our study of cells derived from mice lacking CKS2, we identified a role for CKS2 opposed to its close paralog CKS1. We propose a model in which CKS2 competes with CKS1 for binding to phosphorylated p27 (Figure 7). In this model, CKS2, which lacks the SKP2 binding domain, protects p27 from binding to CKS1 and consequent degradation. The balance of CKS1 versus CKS2 will determine p27 stability and fine-tune its cellular levels.

In the absence of CKS2, p27 stability is reduced, leading to lower cellular p27 levels. The consequences are manifold but mostly attributable to increased activity of cyclin A/CDK2. *Cks2*^−*/*−^ cells showed an overall shortened cell cycle, consistent with reduced levels of a cell cycle inhibitor. S-phase in particular, was affected with an increased average replication fork velocity, accompanied by accumulation of DNA damage. The regulation of replication fork speed by cyclin A/CDK2 activity has not been reported before and warrants further investigation. In addition, we saw an increased incidence of asymmetric forks. This is indicative of fork stalling, which is a likely cause for DNA damage. Regardless, the majority of forks progressed symmetrically. This suggests that the increased fork speed is not the result of an altered replication pausing behavior, but forks indeed progress at a faster rate because of increased cyclin A/CDK2 activity. The reason for increased replication fork velocity could relate to elevated dNTP or histone levels, both of which are controlled by the cell cycle machinery (De Cola et al., 2012; Karnani and Dutta, 2011) and have been shown to limit the speed of fork progression (Poli et al., 2012).

Despite clear signs of an active DNA damage response in *Cks2*^−/−^ cells, we could not detect an expected delay in cell cycle progression. Similarly, exogenous DNA damage at levels sufficient to induce measureable DNA damage response and cell cycle arrest in wild-type cells, failed to delay cell cycle progression in *Cks2*^−/−^ cells. This demonstrates that CKS2 is required as part of the cell cycle machinery to impose checkpoint-dependent cell cycle delays. This could be due to the role of CKS2 in stabilizing p27, which has been implicated in the control of G2 (Nakayama et al., 2004).

p27 binds to both cyclin E/CDK2 and cyclin A/CDK2. We found that only cyclin A/CDK2, but not cyclin E/CDK2, activity was affected in *Cks2*^−/−^ cells (Figure S1B). A recent survey of CDK-associated proteins indicated that CKS proteins interacted mainly with cyclin A-associated CDK complexes but less so with cyclin E complexes (Pagliuca et al., 2011). This could indicate that CKS proteins impact more strongly on cyclin A/CDK2 complexes by controlling their associated p27 levels. During S phase, cyclin E and cyclin A act sequentially to control events in DNA replication. It is therefore possible that, by counteracting SCF^SKP2^-dependent p27 degradation during mid/late S phase when cyclin A expression is highest, CKS2 attenuates cyclin A/CDK2 activity to adjust the dynamics and progression of DNA replication.

### CKS Proteins Determine Cortical Progenitor Cell Fate

To investigate the functional consequences of deregulated p27 levels, we examined the developing brain, as p27 is required for neuronal maturation and migration through the developing cortex in a dose-dependent manner. Cortex formation takes place during embryonic days E11 to E17, when the balance between cortical progenitor self-renewal and cell cycle exit/differentiation determines neuronal fate and laminar formation. Several studies have documented that the timing of cell cycle transition is crucial for neuronal fate determination (Arai et al., 2011; Lange et al., 2009; Calegari and Huttner, 2003; Pilaz et al., 2009).

In *Cks2*^−*/*−^ brains, neuronal differentiation after the first asymmetric cell division was inefficient and, consequently, lamination of the neocortex was affected with fewer mature neurons in the upper layers. Based on the differential expression pattern of CKS1 and CKS2, we propose that the balance between CKS1 and CKS2 govern p27 levels in the developing neuronal layers to control the timing of neuronal differentiation.

### CKS2 in Higher Eukaryotes

Multiple levels of control exist in higher eukaryotes to tightly govern SCF^SKP2^ activity during different phases of the cell cycle. The CKS1/CKS2 axis adds an additional dimension to this control. It remains to be investigated if other potential S phase-specific SCF^SKP2^ substrates are also under the influence of CKS1/CKS2 balance. The replicative and DNA damage response defects observed in *Cks2*^−/−^ cells may suggest a role for CKS2 as a tumor suppressor. Overexpression of CKS2 has been associated with oncogenic roles (Liberal et al., 2012) but this is not mutually exclusive of a potential role as a tumor suppressor. Some genes, including p27, have been shown to act as both oncogenes and tumor suppressors depending on the cellular context (Sicinski et al., 2007). Further investigation that combines the *Cks2*^−*/*−^ mutation with known tumor models would shed insight into this important unexplored area.

## Experimental Procedures

### Generation of *Cks1*^−*/*−^ and *Cks2*^−*/*−^ Murine Models

ES cells containing one disrupted allele by gene trapping for *Cks1* and *Cks2* were obtained from International Gene Trap Consortiums (Nord et al., 2006) (*Cks1*^−*/*−^ mice were constructed from clone S81H1 (Fred Hutchinson Cancer Research Center) and *Cks2*^−*/*−^ mice were constructed using clone DO27C12 (German Gene Trap Consortium). In both cases, the insertion was mapped to the first intron. Mice were generated by standard techniques by the Clinical Science Centre ES/Transgenic facility and maintained in a mixed 129/B6 background. All animal work is in compliance with UK Home Office regulations and institutional review. Western blot using specific antibodies confirmed that no mature proteins at the respective loci were produced in the homozygous knockout mice, respectively.

### Molecular Combing

MEF cells were pulse-labeled for 20 min with 50 μM IdU, quickly washed with PBS and pulse-labeled for another 20 min with 100 μM CldU (Sigma-Aldrich). Cells were then trypsinized, washed, and embedded in low melting agarose plugs (5 × 10^5^ cells/100 μl). After treatment with Proteinase K and Sarkosyl (Sigma-Aldrich), and agarose digestion with β-agarase (New England Biolabs), genomic DNA fibers were combed on silanised coverslips (Microsurfaces, Inc.) and denatured with NaOH. Replicons were visualized using primary antibodies specific for IdU and anti-CldU (Becton Dickenson and Axyll respectively) followed by incubation with AlexaFluor-488 (green) and AlexaFluor-594 (red)-conjugated secondary antibodies (Molecular Probes). Images of replicons were captured using the Epifluorescence Microscope Pathway (Becton Dickinson) with Attovision software. Signals were measured using ImageJ (National Cancer Institute, National Institutes of Health) with custom-made modifications. Replication fork velocities and interorigin distances were calculated using formulas as depicted in Figure 2. Analysis of fork symmetry was performed by measuring the right and left sides of 500 replicons. When the ratio between the two sides was greater than ±0.3, the replicon was labeled as “asymmetrical.”

### Immunoprecipitation and Western Blotting

Cell extracts were prepared in RIPA buffer and sonicated. Soluble fraction was collected after centrifugation. Protein content was determined by colorimetric assay (Biorad). For immunoprecipitation, 1 mg of whole-cell extract was applied to 30 μl of protein A Sepharose slurry (GE Healthcare) together with 5 μg of the specified antibodies. After overnight incubation at 4°C and stringency washes, elution was carried out by boiling in Laemmli buffer. Western blotting was carried out using specific antibodies described in the Supplemental Experimental Procedures.

### siRNA and shRNA

siRNA Smart pools were purchased from Dharmacon and transfected using Hyperfect (QIAGEN) following the manufacturer's instructions. Cells were analyzed 48 hr after transfection.

For the shRNA vector against p53, viral particles were generated by standard transfections with helper plasmids in 293T cells. MEFs were exposed to two rounds of infection of 3 hr and a last one overnight. Selection was carried out after 24 hr with 1 μg/ml puromycin (InVivoGen).

### Cell Cycle Analysis

Cells were harvested by trypsinization, washed in PBS, and fixed in ice-cold 70% ethanol overnight. Cells were then washed twice in PBS, treated with RNaseA, and stained with propidium iodide (PI) for 2 hr at room temperature before being run on a fluorescence-activated cell sorting (FACS) Calibur flow cytometer (Becton Dickinson). For BrdU staining, cells were grown in the presence of BrdU (Sigma-Aldrich) at a final concentration of 1 μM for the durations specified. Harvested cells were treated with 0.1 M borate buffer for 1 hr and then with 3 ml of 2N HCl before labeling with anti-BrdU antibodies (Thermo Fisher: Clone BU-1). Data were analyzed using the CellQuest software (Becton Dickinson).

### Drug Sensitivity Assay

Cells were seeded at a density of 10^4^ per well on a 24-well plate. Six hours later, aphidicolin (Sigma-Aldrich) was added at the concentrations indicated. After 4 days, cells were fixed in 0.5% glutaraldehyde (Sigma-Aldrich) in PBS and stained with Crystal Violet for 20 min, rinsed extensively with running water, and then imaged. Quantification was performed by dissolving the incorporated Crystal Violet in 1% acetic acid and 0.5% SDS and measuring the absorbance at 540 nm. Each point was performed as three experimental replicates.

### Determination of Protein Half-Life

Cells were seeded at ∼60% confluency (7 × 10^5^ cells) in 100-mm plates, synchronized released from nocodazole, and treated with 100 μg/ml cycloheximide for the indicated times. Cells prepared for western blotting.

### p27 Peptide Binding Assay

Recombinant CKS1-flag, or CKS2 or *CKS2* mutant proteins were expressed from *Escherichia coli* and purified by nickel-agarose chromatography in accordance with the manufacturer's instructions (QIAGEN). Biotin-labeled phosphorylated-p27 peptide was synthesized (biotin-Aca-SDG-SPN-AGS-VEQ-T[p]PK-KPG-LRR-RQT-CONH2, Aca = aminocaproic acid) and coupled to a streptavidin affinity column. Recombinant SKP1/SKP2 (Novus Biologicals) were used at a ratio of 7 to 0.4 μM. CKS1-Flag-fusion protein with increasing concentrations of CKS2 or mutant *CKS2* were incubated together with SKP1/2 complex in a 10-μl reaction volume containing 40 mmTris-HCl (pH 7.6), 2 mg/ml bovine serum albumin, 5 mm MgCl_2_, and 1 mm DTT. Incubation was carried out at 4°C for 2 hr, and then the beads were washed four times with 1 ml portions of RIPA buffer. Following elution, samples were subjected to SDS-PAGE and western blotting.

### p27 Cellular Ubiquitylation Assay

Cellular ubiquitylation of p27 was determined as described previously (Montagnoli et al., 1999). Briefly, cells were treated with 10 μM MG132 (Sigma-Aldrich) and denatured in 1% SDS and then diluted in RIPA lysis buffer (50 mM Tris-HCI [pH 7.4], 0.25 M NaCl, 1% Triton X-100, 0.1% SDS, 1 mM EDTA) in the presence of protease inhibitors, phosphatase inhibitors, and 10 mM N-ethylmaleimide (Sigma-Aldrich). A mixture of anti-p27 antibody (3 ug) (BD Biosciences) and anti-phospho-(T187) p27 antibody (3 ug) (Invitrogen) were used for immunoprecipitation. Beads were eluted with 8 M urea and subjected to western blotting.

### In Vitro Kinase Assay

Immunoprecipitates with cyclin A2, cyclin E, or CDK2 prepared from cell lysates were incubated with 5 μg of histone H1 (New England Biolabs) and [γ-^32^P]ATP (PerkinElmer) for 10 min at 30°C. Reaction mixture was resolved by SDS-PAGE, and then phosphorylated histone H1 was analyzed by SDS-PAGE and autoradiography.

### Immunofluorescence and Preparation of Brain Sections

Embryonic day E13.5 brains were dissected and fixed by immersion in 4% paraformaldehyde (PFA) solution overnight. Brains were cryoprotected in 30% sucrose solution, embedded in OCT medium (Thermo Fisher) and frozen in isopentane (Sigma-Aldrich) on dry ice. Coronal cryosections (17 μm) were made, mounted on a glass slide, and stored at −20°C.

Sections were rehydrated in Tris-buffered saline (TBS) for 30 min and incubated in 1% Triton in PBS and 10% normal goat serum (NGS) for 45 min. Primary antibodies were incubated overnight at 4°C in 0.2% Triton and 10% NGS, followed by appropriate secondary antibody and staining with DAPI prior to mounting.

## Supplemental Information

Document S1. Figures S1–S3 and Supplemental Experimental Procedures

## References

[bib1] Anglana M., Apiou F., Bensimon A., Debatisse M. (2003). Dynamics of DNA replication in mammalian somatic cells: nucleotide pool modulates origin choice and interorigin spacing. Cell.

[bib2] Arai Y., Pulvers J.N., Haffner C., Schilling B., Nüsslein I., Calegari F., Huttner W.B. (2011). Neural stem and progenitor cells shorten S-phase on commitment to neuron production. Nat. Commun..

[bib3] Bensimon A., Aebersold R., Shiloh Y. (2011). Beyond ATM: the protein kinase landscape of the DNA damage response. FEBS Lett..

[bib4] Berthet C., Aleem E., Coppola V., Tessarollo L., Kaldis P. (2003). Cdk2 knockout mice are viable. Curr. Biol..

[bib5] Bourne Y., Watson M.H., Hickey M.J., Holmes W., Rocque W., Reed S.I., Tainer J.A. (1996). Crystal structure and mutational analysis of the human CDK2 kinase complex with cell cycle-regulatory protein CksHs1. Cell.

[bib6] Caillava C., Vandenbosch R., Jablonska B., Deboux C., Spigoni G., Gallo V., Malgrange B., Baron-Van Evercooren A. (2011). Cdk2 loss accelerates precursor differentiation and remyelination in the adult central nervous system. J. Cell Biol..

[bib7] Calegari F., Huttner W.B. (2003). An inhibition of cyclin-dependent kinases that lengthens, but does not arrest, neuroepithelial cell cycle induces premature neurogenesis. J. Cell Sci..

[bib8] Conti C., Caburet S., Schurra C., Bensimon A. (2001). Molecular combing. Curr. Protoc. Cytom..

[bib9] Conti C., Saccà B., Herrick J., Lalou C., Pommier Y., Bensimon A. (2007). Replication fork velocities at adjacent replication origins are coordinately modified during DNA replication in human cells. Mol. Biol. Cell.

[bib10] Cotta-Ramusino C., McDonald E.R., Hurov K., Sowa M.E., Harper J.W., Elledge S.J. (2011). A DNA damage response screen identifies RHINO, a 9-1-1 and TopBP1 interacting protein required for ATR signaling. Science.

[bib11] Courbet S., Gay S., Arnoult N., Wronka G., Anglana M., Brison O., Debatisse M. (2008). Replication fork movement sets chromatin loop size and origin choice in mammalian cells. Nature.

[bib12] De Cola A., Bongiorno-Borbone L., Bianchi E., Barcaroli D., Carletti E., Knight R.A., Di Ilio C., Melino G., Sette C., De Laurenzi V. (2012). FLASH is essential during early embryogenesis and cooperates with p73 to regulate histone gene transcription. Oncogene.

[bib13] Dickins R.A., Hemann M.T., Zilfou J.T., Simpson D.R., Ibarra I., Hannon G.J., Lowe S.W. (2005). Probing tumor phenotypes using stable and regulated synthetic microRNA precursors. Nat. Genet..

[bib14] Egan E.A., Solomon M.J. (1998). Cyclin-stimulated binding of Cks proteins to cyclin-dependent kinases. Mol. Cell. Biol..

[bib15] Ge X.Q., Blow J.J. (2010). Chk1 inhibits replication factory activation but allows dormant origin firing in existing factories. J. Cell Biol..

[bib16] Hao B., Zheng N., Schulman B.A., Wu G., Miller J.J., Pagano M., Pavletich N.P. (2005). Structural basis of the Cks1-dependent recognition of p27(Kip1) by the SCF(Skp2) ubiquitin ligase. Mol. Cell.

[bib17] Holic R., Kukalev A., Lane S., Andress E.J., Lau I., Yu C.W., Edelmann M.J., Kessler B.M., Yu V.P. (2010). Cks1 activates transcription by binding to the ubiquitylated proteasome. Mol. Cell. Biol..

[bib18] Jablonska B., Aguirre A., Vandenbosch R., Belachew S., Berthet C., Kaldis P., Gallo V. (2007). Cdk2 is critical for proliferation and self-renewal of neural progenitor cells in the adult subventricular zone. J. Cell Biol..

[bib19] Karnani N., Dutta A. (2011). The effect of the intra-S-phase checkpoint on origins of replication in human cells. Genes Dev..

[bib20] Kawabata T., Luebben S.W., Yamaguchi S., Ilves I., Matise I., Buske T., Botchan M.R., Shima N. (2011). Stalled fork rescue via dormant replication origins in unchallenged S phase promotes proper chromosome segregation and tumor suppression. Mol. Cell.

[bib21] Kõivomägi M., Valk E., Venta R., Iofik A., Lepiku M., Balog E.R., Rubin S.M., Morgan D.O., Loog M. (2011). Cascades of multisite phosphorylation control Sic1 destruction at the onset of S phase. Nature.

[bib22] Lange C., Huttner W.B., Calegari F. (2009). Cdk4/cyclinD1 overexpression in neural stem cells shortens G1, delays neurogenesis, and promotes the generation and expansion of basal progenitors. Cell Stem Cell.

[bib23] Liberal V., Martinsson-Ahlzén H.S., Liberal J., Spruck C.H., Widschwendter M., McGowan C.H., Reed S.I. (2012). Cyclin-dependent kinase subunit (Cks) 1 or Cks2 overexpression overrides the DNA damage response barrier triggered by activated oncoproteins. Proc. Natl. Acad. Sci. USA.

[bib24] Martinsson-Ahlzén H.S., Liberal V., Grünenfelder B., Chaves S.R., Spruck C.H., Reed S.I. (2008). Cyclin-dependent kinase-associated proteins Cks1 and Cks2 are essential during early embryogenesis and for cell cycle progression in somatic cells. Mol. Cell. Biol..

[bib25] Merrick K.A., Larochelle S., Zhang C., Allen J.J., Shokat K.M., Fisher R.P. (2008). Distinct activation pathways confer cyclin-binding specificity on Cdk1 and Cdk2 in human cells. Mol. Cell.

[bib26] Merrick K.A., Wohlbold L., Zhang C., Allen J.J., Horiuchi D., Huskey N.E., Goga A., Shokat K.M., Fisher R.P. (2011). Switching Cdk2 on or off with small molecules to reveal requirements in human cell proliferation. Mol. Cell.

[bib27] Montagnoli A., Fiore F., Eytan E., Carrano A.C., Draetta G.F., Hershko A., Pagano M. (1999). Ubiquitination of p27 is regulated by Cdk-dependent phosphorylation and trimeric complex formation. Genes Dev..

[bib28] Morris M.C., Kaiser P., Rudyak S., Baskerville C., Watson M.H., Reed S.I. (2003). Cks1-dependent proteasome recruitment and activation of CDC20 transcription in budding yeast. Nature.

[bib29] Nakayama K., Nagahama H., Minamishima Y.A., Miyake S., Ishida N., Hatakeyama S., Kitagawa M., Iemura S., Natsume T., Nakayama K.I. (2004). Skp2-mediated degradation of p27 regulates progression into mitosis. Dev. Cell.

[bib30] Nguyen L., Besson A., Heng J.I., Schuurmans C., Teboul L., Parras C., Philpott A., Roberts J.M., Guillemot F. (2006). p27kip1 independently promotes neuronal differentiation and migration in the cerebral cortex. Genes Dev..

[bib31] Nguyen L., Besson A., Roberts J.M., Guillemot F. (2006). Coupling cell cycle exit, neuronal differentiation and migration in cortical neurogenesis. Cell Cycle.

[bib32] Nord A.S., Chang P.J., Conklin B.R., Cox A.V., Harper C.A., Hicks G.G., Huang C.C., Johns S.J., Kawamoto M., Liu S. (2006). The International Gene Trap Consortium Website: a portal to all publicly available gene trap cell lines in mouse. Nucleic Acids Res..

[bib33] Ortega S., Prieto I., Odajima J., Martín A., Dubus P., Sotillo R., Barbero J.L., Malumbres M., Barbacid M. (2003). Cyclin-dependent kinase 2 is essential for meiosis but not for mitotic cell division in mice. Nat. Genet..

[bib34] Pagano M., Pepperkok R., Lukas J., Baldin V., Ansorge W., Bartek J., Draetta G. (1993). Regulation of the cell cycle by the cdk2 protein kinase in cultured human fibroblasts. J. Cell Biol..

[bib35] Pagliuca F.W., Collins M.O., Lichawska A., Zegerman P., Choudhary J.S., Pines J. (2011). Quantitative proteomics reveals the basis for the biochemical specificity of the cell-cycle machinery. Mol. Cell.

[bib36] Patra D., Wang S.X., Kumagai A., Dunphy W.G. (1999). The xenopus Suc1/Cks protein promotes the phosphorylation of G(2)/M regulators. J. Biol. Chem..

[bib37] Pilaz L.J., Patti D., Marcy G., Ollier E., Pfister S., Douglas R.J., Betizeau M., Gautier E., Cortay V., Doerflinger N. (2009). Forced G1-phase reduction alters mode of division, neuron number, and laminar phenotype in the cerebral cortex. Proc. Natl. Acad. Sci. USA.

[bib38] Poli J., Tsaponina O., Crabbé L., Keszthelyi A., Pantesco V., Chabes A., Lengronne A., Pasero P. (2012). dNTP pools determine fork progression and origin usage under replication stress. EMBO J..

[bib39] Polo S.E., Jackson S.P. (2011). Dynamics of DNA damage response proteins at DNA breaks: a focus on protein modifications. Genes Dev..

[bib40] Reynard G.J., Reynolds W., Verma R., Deshaies R.J. (2000). Cks1 is required for G(1) cyclin-cyclin-dependent kinase activity in budding yeast. Mol. Cell. Biol..

[bib41] Santamaría D., Barrière C., Cerqueira A., Hunt S., Tardy C., Newton K., Cáceres J.F., Dubus P., Malumbres M., Barbacid M. (2007). Cdk1 is sufficient to drive the mammalian cell cycle. Nature.

[bib42] Seiler J.A., Conti C., Syed A., Aladjem M.I., Pommier Y. (2007). The intra-S-phase checkpoint affects both DNA replication initiation and elongation: single-cell and -DNA fiber analyses. Mol. Cell. Biol..

[bib43] Sicinski P., Zacharek S., Kim C. (2007). Duality of p27Kip1 function in tumorigenesis. Genes Dev..

[bib44] Sitry D., Seeliger M.A., Ko T.K., Ganoth D., Breward S.E., Itzhaki L.S., Pagano M., Hershko A. (2002). Three different binding sites of Cks1 are required for p27-ubiquitin ligation. J. Biol. Chem..

[bib45] Spruck C., Strohmaier H., Watson M., Smith A.P., Ryan A., Krek T.W., Reed S.I. (2001). A CDK-independent function of mammalian Cks1: targeting of SCF(Skp2) to the CDK inhibitor p27Kip1. Mol. Cell.

[bib46] Spruck C.H., de Miguel M.P., Smith A.P., Ryan A., Stein P., Schultz R.M., Lincoln A.J., Donovan P.J., Reed S.I. (2003). Requirement of Cks2 for the first metaphase/anaphase transition of mammalian meiosis. Science.

[bib47] Tane S., Chibazakura T. (2009). Cyclin A overexpression induces chromosomal double-strand breaks in mammalian cells. Cell Cycle.

[bib48] Todaro G.J., Green H. (1963). Quantitative studies of the growth of mouse embryo cells in culture and their development into established lines. J. Cell Biol..

[bib49] van Zon W., Ogink J., ter Riet B., Medema R.H., te Riele H., Wolthuis R.M. (2010). The APC/C recruits cyclin B1-Cdk1-Cks in prometaphase before D box recognition to control mitotic exit. J. Cell Biol..

[bib50] Westbrook L., Manuvakhova M., Kern F.G., Estes N.R., Ramanathan H.N., Thottassery J.V. (2007). Cks1 regulates cdk1 expression: a novel role during mitotic entry in breast cancer cells. Cancer Res..

[bib51] Wheeler L.W., Lents N.H., Baldassare J.J. (2008). Cyclin A-CDK activity during G1 phase impairs MCM chromatin loading and inhibits DNA synthesis in mammalian cells. Cell Cycle.

[bib52] Wilsker D., Petermann E., Helleday T., Bunz F. (2008). Essential function of Chk1 can be uncoupled from DNA damage checkpoint and replication control. Proc. Natl. Acad. Sci. USA.

[bib53] Wolthuis R., Clay-Farrace L., van Zon W., Yekezare M., Koop L., Ogink J., Medema R., Pines J. (2008). Cdc20 and Cks direct the spindle checkpoint-independent destruction of cyclin A. Mol. Cell.

[bib54] Yao Z.P., Zhou M., Kelly S.E., Seeliger M.A., Robinson C.V., Itzhaki L.S. (2006). Activation of ubiquitin ligase SCF(Skp2) by Cks1: insights from hydrogen exchange mass spectrometry. J. Mol. Biol..

[bib55] Yu V.P., Baskerville C., Grünenfelder B., Reed S.I. (2005). A kinase-independent function of Cks1 and Cdk1 in regulation of transcription. Mol. Cell.

