# Supplementary material for: The CDK Subunit CKS2 Counteracts CKS1 to Control Cyclin A/CDK2 Activity in Maintaining Replicative Fidelity and Neurodevelopment
Source: Dev Cell. 2012 Aug 14;23(2):356–70. doi: 10.1016/j.devcel.2012.06.018 (PMC3898080; doi:10.1016/j.devcel.2012.06.018)
Supplement: Document S1. Figures S1–S3 and Supplemental Experimental Procedures [file mmc1.pdf]

Developmental Cell, Volume *23*

Supplemental Information

The CDK Subunit CKS2 Counteracts CKS1 to Control Cyclin A/CDK2 Activity in Maintaining Replicative Fidelity and Neurodevelopment

Mattia Frontini, Alexander Kukalev, Elisabetta Leo, Yiu-Ming Ng, Marcella Cervantes, Chi-Wai Cheng, Roman Holic, Dirk Dormann, Eric Tse, Yves Pommier, and Veronica Yu

Inventory of Supplemental Information

Supplementary Figures:

Figure S1 (related to Figure 4):

(A, B) Roscovitine reverses *Cks2*$^{-/-}$ defects

(C) Cyclin E/CDK2 activity is unaffected by CKS2

Figure S2 (related to Figure 5):

Over-expression of p27 reverses hyperactivity of CyclinA/CDK2 in *Cks2*$^{-/-}$ cells.

Figure S3 (related to Figure 6):

(A) Expression pattern of Cks1 and Cks2 in the fetal brain

(B) Expression of p27 in *Cks1*$^{-/-}$ fetal brain

(C) Cell cycle transition in *Cks1*$^{-/-}$ and *Cks2*$^{-/-}$ fetal brains

Supplementary Experimental Procedures

Supplementary References

Figure S1

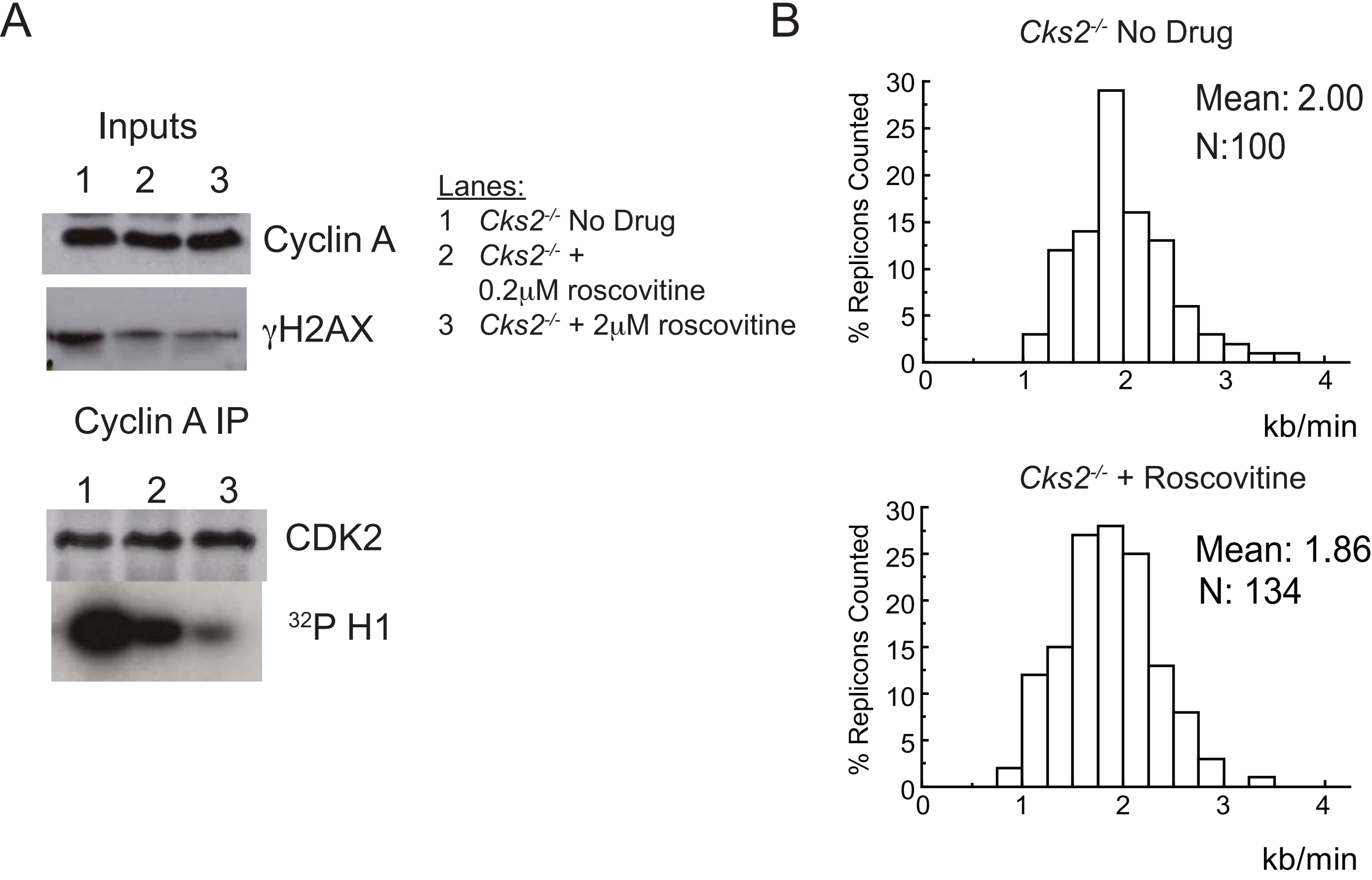

A
Inputs
1 2 3
Cyclin A
γH2AX
Cyclin A IP
1 2 3
CDK2
32P H1
Lanes:
1 Cks2-/- No Drug
2 Cks2-/- + 0.2μM roscovitine
3 Cks2-/- + 2μM roscovitine
B
Cks2-/- No Drug
Mean: 2.00
N:100
% Replicons Counted
kb/min
Cks2-/- + Roscovitine
Mean: 1.86
N: 134
% Replicons Counted
kb/min

Figure S1

C

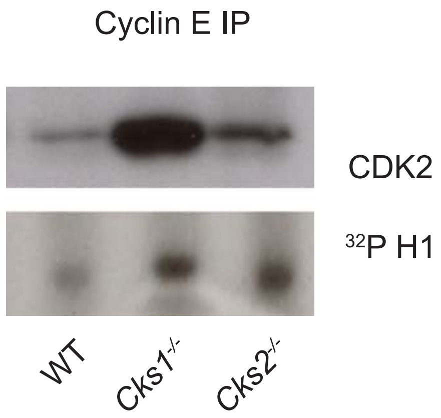

Cyclin E IP
CDK2
32P H1
WT
Cks1-/-
Cks2-/-

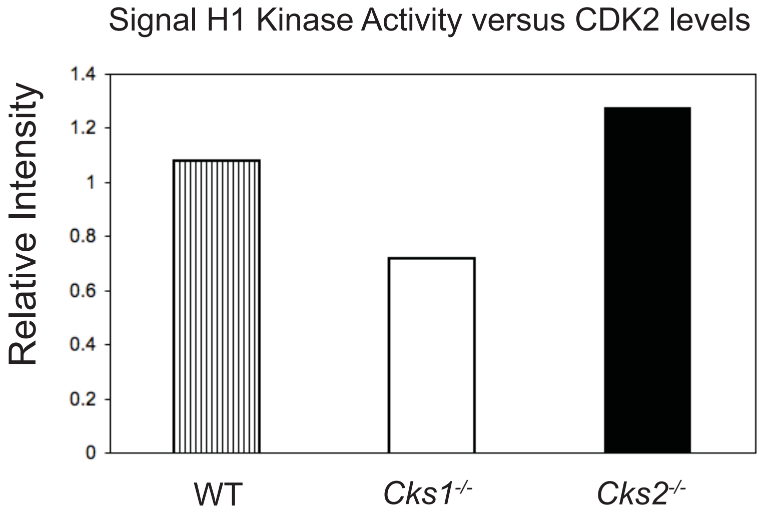

Signal H1 Kinase Activity versus CDK2 levels
Relative Intensity
1.4
1.2
1
0.8
0.6
0.4
0.2
0
WT
Cks1-/-
Cks2-/-

Figure S1 (related to Figure 4)**:**
Roscovitine reverses *Cks2$^{-/-}$* defects
(A) *Cks2$^{-/-}$* cells were co-cultured with increasing concentrations of the CDK inhibitor roscovitine and subjected to immunoprecipitation against Cyclin A. Input samples were blotted against Cyclin A and γH2AX. Immunoprecipitated samples were subjected to *in vitro* H1 Kinase analysis to demonstrate relative CyclinA/CDK2 activity before and after drug treatment.
(B) Molecular combing of DNA fibers from *Cks2$^{-/-}$* MEFs in the presence or absence of 0.2μM of roscovitine. At least 100 fibres were counted per sample (represented by N=) and mean fork velocity was calculated by dividing the total length of two sequential IdU and CldU signals by pulse time (20 minutes each).
Cyclin E/CDK2 activity is unaffected by CKS2
(C) Cyclin E was immunoprecipitated from wild type, *Cks1$^{-/-}$* and *Cks2$^{-/-}$* MEFs. The associated CDK2 levels were analyzed by Western blotting and associated kinase activity measured using histone H1 as the substrate. Kinase activity normalized to CDK2 levels is presented. Cyclin E levels are known to be increased in *Cks1$^{-/-}$* cells (Spruck et al., 2001), to compensate for increased p27 levels. In contrast, little change in the levels or activity of Cyclin E was found in *Cks2$^{-/-}$* cells, compared to the wild type control.

Figure S2

A

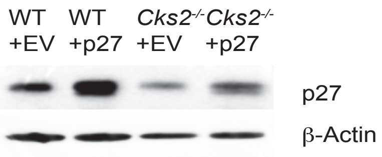

B

WT + EV	Reading	Minus blank	ADP produced (nmol)
Blank (no Histone H1 substrate)	4321929		0.1687852
Sample	5249327	927398	
**WT + p27**			
Blank	3028086		0.0939164
Sample	3581140	553054	
***Cks2$^{-/-}$* + EV**			
Blank	2890022		0.652933
Sample	6238159	3348137	
***Cks2$^{-/-}$* + p27**			
Blank	3038541		0.1837746
Sample	4040886	1002345	

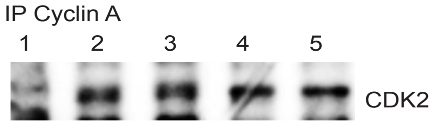

Lanes:
1 Mock IP
2 WT + EV
3 WT + p27
4 *Cks2$^{-/-}$* + EV
5 *Cks2$^{-/-}$* + p27

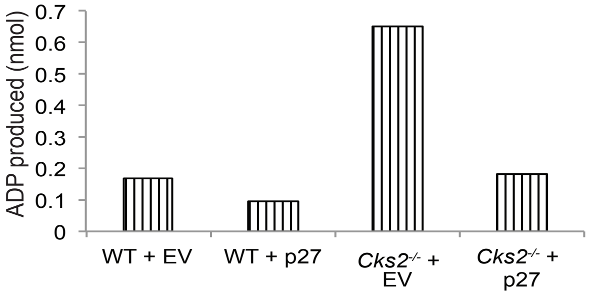

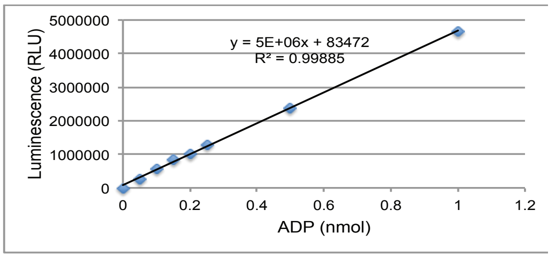

Positive control: 0.1 nmol ADP was produced per ng recombinant CyclinA/Cdk2 in this experiment

Figure S2 (related to Figure 5): **Over-expression of p27 reverses hyperactivity of CyclinA/CDK2 in *Cks2$^{-/-}$* cells**

(A) p27 was transiently over-expressed in wild-type (WT) or *Cks2$^{-/-}$* MEFs. Western blot against p27 demonstrating the level of p27 expression in whole cell lysate in cells transfected with either empty vector (EV) or vector expressing wild-type p27.

(B) Wild-type (WT) or *Cks2$^{-/-}$* MEFs from (A) were subjected to immunoprecipitation against Cyclin A. An *in vitro* fluorescence-based kinase assay was applied to quantify CDK2 activity after p27 over-expression. Briefly, 1mg cell lysate was immune-precipitated using 5µg of Anti-Cyclin A2 antibody (Santa Cruz sc-596) linked to Protein A/G-Sepharose (Invitrogen, Carlsbad, CA, USA). Following overnight incubation at 4°C with and two washes with RIPA buffer, beads were washed twice with 1X kinase buffer (40 mM Tris-HCl, pH 7.5, 20 mM $MgCl_2$ and 0.1 mg/ml BSA) and resuspended in 25µl of 2X kinase buffer. Resuspended beads (10µl) were incubated with 5 µg of histone H1 and 50 µM of ATP at 30°C for 60 min. Kinase activity was determined by the ADP-Glo^{TM} Kinase assay (Promega, Madison, WI, USA) according to the manufacturer's instructions.

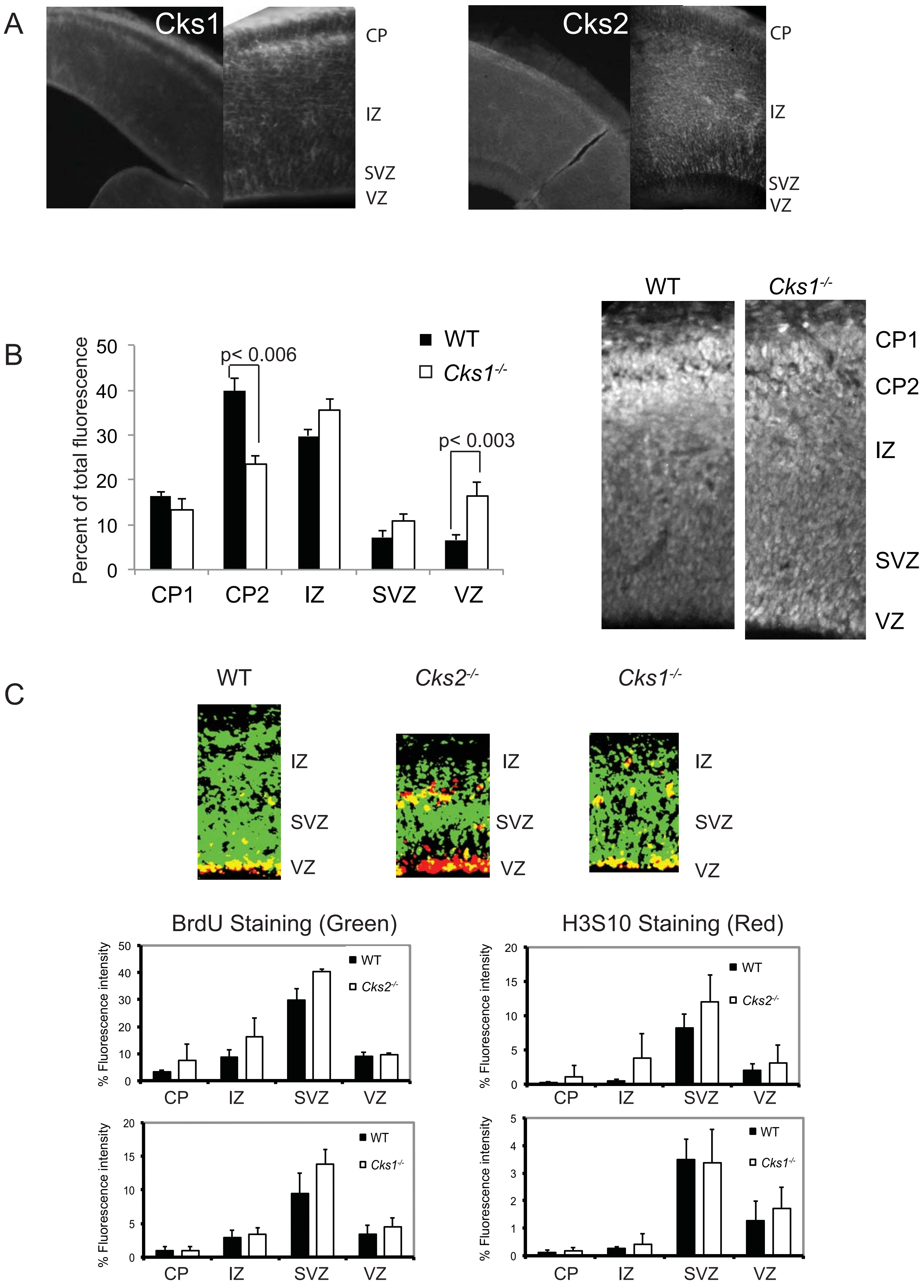
A
Cks1
CP
IZ
SVZ
VZ
Cks2
CP
IZ
SVZ
VZ
B
Percent of total fluorescence
50
40
30
20
10
0
p< 0.006
p< 0.003
WT
Cks1-/-
CP1
CP2
IZ
SVZ
VZ
WT
Cks1-/-
CP1
CP2
IZ
SVZ
VZ
C
WT
Cks2-/-
Cks1-/-
IZ
SVZ
VZ
BrdU Staining (Green)
H3S10 Staining (Red)
% Fluorescence intensity
WT
Cks2-/-
Cks1-/-
CP
IZ
SVZ
VZ

Figure S3 (related to Figure 6):

(A) Expression pattern of Cks1 and Cks2 in the fetal brain
E13.5 cortical section from a representative heterozygote *Cks1*$^{+/-}$ mouse (left) and a heterozygote *Cks2*$^{+/-}$ mouse (right). Fetal brain was PFA fixed and stained using an anti-β-galactosidase antibody (Abcam ab9361) as described (Krechowec et al., 2012). CP: Cortical plate; IZ: Immediate zone; SVZ: Subventricular zone; VZ: Ventricular zone

(B) Immunohistochemistry of p27
Quantification of p27 signals in E13.5 *Cks1*$^{-/-}$ cortical sections were compared to wild-type (WT). Representative images (right). The respective intensity of p27 staining was normalized against the signal of DAPI in each sample. Each data point is an average of 3 experimental replicates (3 fetal brains of equivalent coronal sections). Error bars represent standard deviation.

(C) Cell cycle transition in *Cks1*$^{-/-}$ and *Cks2*$^{-/-}$ fetal brains
Double-labeled immunohistochemistry with BrdU and phospho-histone H3. E13.5 litters were pulsed-labeled by injection of BrdU into the pregnant female 24 hours prior to sacrifice according to established protocols (Arai et al., 2011; Nowakowski et al., 1989). Fetal brains were fixed and sectioned and stained with anti-BrdU antibodies (green) to demonstrate cells which entered S phase during the 24 hour period. The mitotic phospho-histone H3 antibody was used to label cells in G2/M phase of the cell cycle (red). Cells stained positive for both markers (yellow) represent cells which have entered S phase during the 24 hour period and subsequently passed through mitosis (representative images in wild-type (WT), *Cks2*$^{-/-}$ and *Cks1*$^{-/-}$ brains, top). Bottom: graphs quantifying average of 3 experimental replicates. Error bars represent standard deviation.

Supplemental Experimental Procedures

Cell Culture

MEFs were generated from heterozygous crosses for both strains. WT and *Cks* knockout MEFs obtained from littermates were either spontaneously immortalized following a 3T3 protocol or immortalized at first passage using an shRNA against p53 (Dickins et al., 2005). MEFs were maintained in DMEM 4.5g/l glucose (Invitrogen) supplemented with 10% FBS (Sigma), Pen/Strep 1%, Glutamine 1% and nonessential amino acids (PAA) in a 5% CO_2 atmosphere at 37ºC.

Live cell imaging

Multiple site time-lapse recordings of cells growing in 35mm glass bottom dishes (MatTek Corp.) were carried out on two different systems. On a Deltavision Core system, images were recorded with an Olympus 20x/0.5NA UPLFLN objective lens and a Coolsnap HQ (Photometrics) camera. The other system consisted of a Zeiss Axiovert 200 microscope with 10x/0.3NA Plan-NEOFLUAR Ph1 objective, a MS-2000 motorized stage (ASI) and an ORCA-ER CCD camera (Hamamatsu) controlled by Volocity acquisition software (version 5.1, PerkinElmer). Environmental chambers (Solent Scientific) maintained temperature and CO_2 levels. Images were recorded at 3-minute intervals. One cell cycle was calculated as the time-lapse between 2 successive anaphases (as in shRNA immortalized MEFs) or 2 successive cytokineses (as in spontaneously immortalized MEFs, see Figures legends). Each sample was filmed for the duration of at least 2.5 consecutive cell cycles. Mean division time was calculated from at least 50 divisions for each genotype.

Antibodies used in this study

Western blotting and immunoprecipitation

CKS1/2 (Santa Cruz: FL-79 sc-6238); CDK2 (Santa Cruz M2 sc-163); ATM (Cell Signaling #2873); p-ATM (Cell Signaling ATM S1981 #5883); CHK2 (Cell Signaling #2662); p-CHK2 (Cell Signaling #2665); CHK1 (Abcam ab47574); p-CHK1 (Abcam S317 ab2834); cyclin A2 (Santa Cruz C-19 sc-596); cyclin E (Upstate 07-687); cyclin B1 (V152 ab72); γH2AX (p-S139) (Millipore JBW301 05-636); p27 monoclonal antibody (BD Bioscience 610241); p27 polyclonal antibody (Santa Cruz C19 sc-528); PCNA (Cell Signaling PC10); tubulin (Sigma T6557); anti-FLAG antibody (Clone M2, F3165, Sigma-Aldrich); anti-ubiquitin antibody (Abcam, ab19247).

Immunohistochemistry

Brain sections: Anti-Pax6 (Covance PRB-278P, 1:100), anti-Tbr1 (Abcam ab31940, 1:300) and anti-Tbr2 (Abcam ab23345, 1:300).

For staining of γH2AX foci, MEFs were culture on coverslips, fixed and permeabilised according to Paull *et al*. (Paull et al., 2000). Anti-γH2AX antibody (Millipore Clone JBW301) was used at a dilution of 1:200.

Supplemental References

Arai, Y., Pulvers, J.N., Haffner, C., Schilling, B., Nusslein, I., Calegari, F., and Huttner, W.B. (2011). Neural stem and progenitor cells shorten S-phase on commitment to neuron production. Nature communications *2*, 154.

Dickins, R.A., Hemann, M.T., Zilfou, J.T., Simpson, D.R., Ibarra, I., Hannon, G.J., and Lowe, S.W. (2005). Probing tumor phenotypes using stable and regulated synthetic microRNA precursors. Nat Genet 37, 1289-1295.

Krechowec, S.O., Burton, K.L., Newlaczyl, A.U., Nunn, N., Vlatkovic, N., and Plagge, A. (2012). Postnatal changes in the expression pattern of the imprinted signalling protein XLalphas underlie the changing phenotype of deficient mice. PloS one *7*, e29753.

Paull, T.T., Rogakou, E.P., Yamazaki, V., Kirchgessner, C.U., Gellert, M., and Bonner, W.M. (2000). A critical role for histone H2AX in recruitment of repair factors to nuclear foci after DNA damage. Current biology : CB *10*, 886-895.

Nowakowski, R.S., Lewin, S.B., and Miller, M.W. (1989). Bromodeoxyuridine immunohistochemical determination of the lengths of the cell cycle and the DNA-synthetic phase for an anatomically defined population. Journal of neurocytology *18*, 311-318.

Spruck, C., Strohmaier, H., Watson, M., Smith, A.P., Ryan, A., Krek, T.W., and Reed, S.I. (2001). A CDK-independent function of mammalian Cks1: targeting of SCF(Skp2) to the CDK inhibitor p27Kip1. Molecular cell *7*, 639-650.
